# CEP162 deficiency causes human retinal degeneration and reveals a dual role in ciliogenesis and neurogenesis

**DOI:** 10.1172/JCI161156

**Published:** 2023-04-17

**Authors:** Nafisa Nuzhat, Kristof Van Schil, Sandra Liakopoulos, Miriam Bauwens, Alfredo Dueñas Rey, Stephan Käseberg, Melanie Jäger, Jason R. Willer, Jennifer Winter, Hanh M. Truong, Nuria Gruartmoner, Mattias Van Heetvelde, Joachim Wolf, Robert Merget, Sabine Grasshoff-Derr, Jo Van Dorpe, Anne Hoorens, Heidi Stöhr, Luke Mansard, Anne-Françoise Roux, Thomas Langmann, Katharina Dannhausen, David Rosenkranz, Karl M. Wissing, Michel Van Lint, Heidi Rossmann, Friederike Häuser, Peter Nürnberg, Holger Thiele, Ulrich Zechner, Jillian N. Pearring, Elfride De Baere, Hanno J. Bolz

**Affiliations:** 1 Department of Cell and Developmental Biology, University of Michigan, Ann Arbor, Michigan, USA.; 2 Department of Biomolecular Medicine, Ghent University, Ghent, Belgium.; 3 Center for Medical Genetics, Ghent University Hospital, Ghent, Belgium.; 4 Cologne Image Reading Center, Department of Ophthalmology, University Hospital of Cologne, Cologne, Germany.; 5 Department of Ophthalmology, Goethe University, Frankfurt, Germany.; 6 Institute of Human Genetics, University Medical Center Mainz, Mainz, Germany.; 7 Department of Ophthalmology, Justus-Liebig-University Giessen, Giessen, Germany.; 8 Augenarztpraxis Bad Brückenau, Bad Brückenau, Germany.; 9 Department of Ophthalmology and; 10 Cell and Molecular Biology Program, University of Michigan, Ann Arbor, Michigan, USA.; 11 Department of Radiology and; 12 Department of Pediatric Surgery, Bürgerhospital, Frankfurt am Main, Germany.; 13 Department of Diagnostic Sciences, Ghent University, Ghent, Belgium.; 14 Department of Pathology, Ghent University Hospital, Ghent, Belgium.; 15 Institute of Human Genetics, University of Regensburg, Regensburg, Germany.; 16 Laboratoire de Génétique Moléculaire, CHU de Montpellier, Université de Montpellier, Montpellier, France.; 17 Laboratory for Experimental Immunology of the Eye, Department of Ophthalmology, Faculty of Medicine and University Hospital Cologne, Cologne, Germany.; 18 Senckenberg Centre for Human Genetics, Frankfurt am Main, Germany.; 19 Department of Nephrology and; 20 Department of Ophthalmology, Brussels University Hospital, Jette, Belgium.; 21 Institute of Clinical Chemistry and Laboratory Medicine, University Medical Center Mainz, Mainz, Germany.; 22 Cologne Center for Genomics (CCG), University of Cologne, Faculty of Medicine and University Hospital Cologne, Cologne, Germany.; 23 Center for Molecular Medicine Cologne, University of Cologne, Cologne, Germany.; 24 Institute of Human Genetics, University Hospital of Cologne, Cologne, Germany.

**Keywords:** Cell Biology, Genetics, Genetic diseases, Molecular genetics, Retinopathy

## Abstract

Defects in primary or motile cilia result in a variety of human pathologies, and retinal degeneration is frequently associated with these so-called ciliopathies. We found that homozygosity for a truncating variant in CEP162, a centrosome and microtubule-associated protein required for transition zone assembly during ciliogenesis and neuronal differentiation in the retina, caused late-onset retinitis pigmentosa in 2 unrelated families. The mutant CEP162-E646R*5 protein was expressed and properly localized to the mitotic spindle, but it was missing from the basal body in primary and photoreceptor cilia. This impaired recruitment of transition zone components to the basal body and corresponded to complete loss of CEP162 function at the ciliary compartment, reflected by delayed formation of dysmorphic cilia. In contrast, shRNA knockdown of *Cep162* in the developing mouse retina increased cell death, which was rescued by expression of CEP162-E646R*5, indicating that the mutant retains its role for retinal neurogenesis. Human retinal degeneration thus resulted from specific loss of the ciliary function of CEP162.

## Graphical abstract

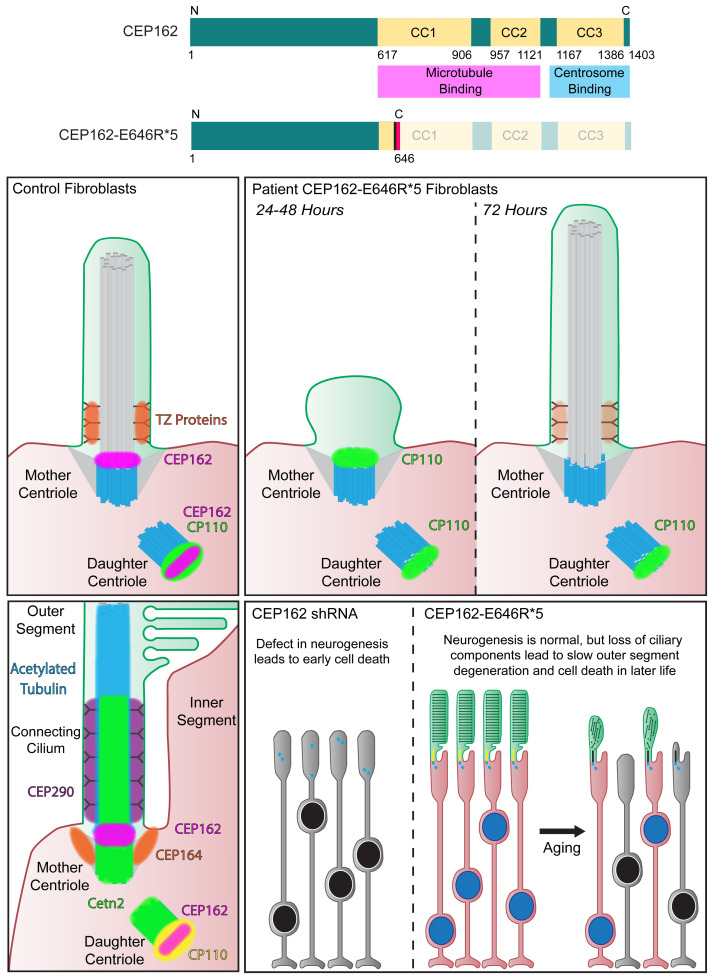

## Introduction

Primary cilia are sensory organelles that protrude from the cell surface to detect extracellular signals that regulate cellular physiology. Cilia are highly dynamic microtubule-based organelles that are nucleated from the mother centriole and assembled and disassembled in each round of the cell cycle. The complex process of cilium assembly, maintenance, and disassembly is estimated to require thousands of genes, with nearly 700 proteins confirmed to be localized to the cilium (1) and many more implicated in ciliary function. Dysfunction of ciliary genes and proteins is associated with a wide range of human disorders. These ciliopathies can involve virtually any organ, though they affect the retina quite frequently. Proteins with established roles in cilia may also participate in other cellular processes. For example, OFD1, a centrosomal protein of the basal body implicated in isolated retinitis pigmentosa (RP) and different syndromes (2, 3), is also involved in chromatin remodeling (4) and cell cycle progression (5).

*CEP162* was originally cloned from quail retinal neurons and designated quail neuroretina 1 (QN1) (6). Inhibition of QN1 during retinal development led to defective mitosis and differentiation, suggesting that it is involved in withdrawal from the cell cycle, so QN1 was postulated to play a role in neuronal quiescence (7, 8). QN1 is orthologous to human KIAA1009/CEP162 (9), a protein that binds to microtubule spindles during mitosis and localizes to the distal ends of centrioles in postmitotic cells (8, 10). Loss of CEP162 has been shown to arrest ciliogenesis at the stage of transition zone (TZ) assembly and to cause a ciliopathy phenotype in zebrafish (10). Despite its importance for cilia formation, no pathogenic *CEP162* variants have been reported in human disease, and its role in the retina remains unknown.

Here, we show that homozygosity for a frameshift variant in *CEP162* causes late-onset RP in 2 unrelated families. We found that the truncated CEP162 is unable to localize to the basal body. Absence of CEP162 from the primary cilium resulted in a loss of some TZ components and delayed formation of dysmorphic cilia. However, truncated CEP162 maintained its capability to bind microtubules and localized to the mitotic spindle, suggesting that it could retain function in neuronal cell division. Indeed, we found that increased cell death in the developing mouse retina with loss of *Cep162* expression could be restored not only by full-length, but also by truncated CEP162, which is unable to localize to the basal body in these cells. Thus, specific loss of CEP162 function at the primary cilium is likely the primary cause of the late-onset human retinal ciliopathy.

## Results

### Late-onset RP in 2 unrelated patients.

**Figure 1:**
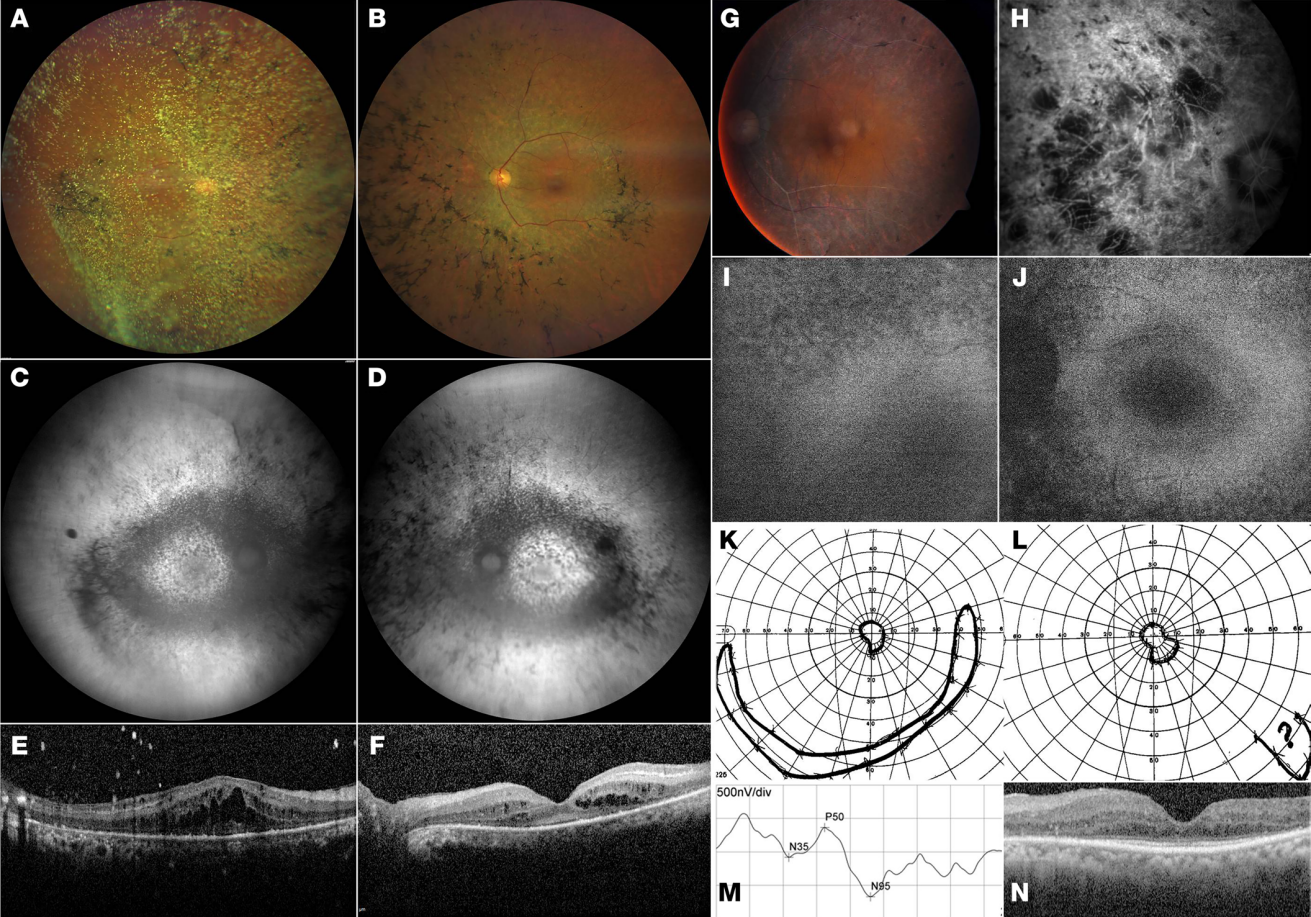
Ophthalmological data of patients 1 and 2 with RP. Ophthalmological data of patient 1 (**A**–**F**) and patient 2 (**G**–**N**) with RP are presented throughout (**A**) Right eye (OD) and (**B**) left eye (OS) with pale optic disc, narrow vessels, and bone spicule pigmentation on color fundus photography. (**C** and **D**) Granular decreased autofluorescence throughout the posterior pole on blue light autofluorescence. (**E** and **F**) Spectral domain optical coherence tomography (SDOCT): Cystoid spaces in the inner and outer nuclear layer and thinning of the outer nuclear layer with sparing of the central fovea. (**G**) Color fundus photography of the left eye: Pale optic disc, narrow vessels with pronounced sheathing giving a white, pseudothrombotic, aspect, bone spicule pigmentation. (**H**) Fluorescein angiography of the right eye: Strong atrophy of the outer retina and few intraretinal pigment migrations. Autofluorescence from (**I**) right eye and (**J**) left eye: bull’s eye aspect of the macula. Goldmann visual fields for (**K**) left eye and (**L**) right eye: constriction of < 10°. (**M**) Pattern ERG of left eye: Reduced macular activity (visual acuity 3/10). No responses for the right eye. (**N**) Optical coherence tomography: Absence of the outer nuclear layer beyond the macula.

Patient 1’s loss of visual acuity was noted at 56 years of age. At 60 years, RP was diagnosed. Night blindness had presumably existed for years. There is now noticeable photophobia. At the age of 67 years, refraction revealed hyperopia and astigmatism in both eyes with +6.25 Sphere (sph), –1.25 Cylinder (cyl)/71° in the right eye and +2.75 sph, –1.00 cyl/58° in the left eye. Color fundus photography, blue light autofluorescence, and spectral domain optical coherence tomography were compatible with RP, including pale optic disc, narrow vessels, bone spicule pigmentation, and thinning of the outer nuclear layer (Figure 1, A–F). Best corrected visual acuity (BCVA) was 20/500 for both eyes, based on the Snellen chart, and visual fields were constricted. There was *synchysis scintillans* in the right vitreous. Patient 2’s loss of visual acuity was reported since the fifth decade. At the age of 69 years, RP was diagnosed. BCVA was light perception in the right eye (OD) and 4/10 in the left eye (OS) and visual fields were constricted less than 10°. Color fundus photography showed a pale optic disk, narrow vessels with sheathing, and limited bone spicule pigmentation. Fluorescein angiography displayed strong atrophy of the outer retina and few intraretinal pigment migrations. Optical coherence tomography showed an absence of the outer retina, except in the fovea. On blue light autofluorescence, a bull’s eye aspect was observed. A pattern electroretinogram (ERG) showed an absent response (OD) and reduced macular function (OS) (Figure 1, G–N). A full-field ERG could not be conducted. Patient 2 was followed at the diabetes clinic for 30 years; no islet cell autoantibodies were detected. There was a low normal production of C-peptide (0.20 nmol/L.; reference 0.29–0.99 nmol/L). He received medication for chronic renal insufficiency due to diabetic nephropathy. Abdominal ultrasound and CT showed no kidney abnormalities (Supplemental Figure 1; supplemental material available online with this article; https://doi.org/10.1172/JCI161156DS1), but a lipomatous aspect of the pancreas. Patient 2 suffered from coronary main stem stenosis (50%–59%; noninvasive treatment) and died at the age of 74.

### Homozygous CEP162 frameshift variant in both unrelated RP patients.

**Figure 2:**
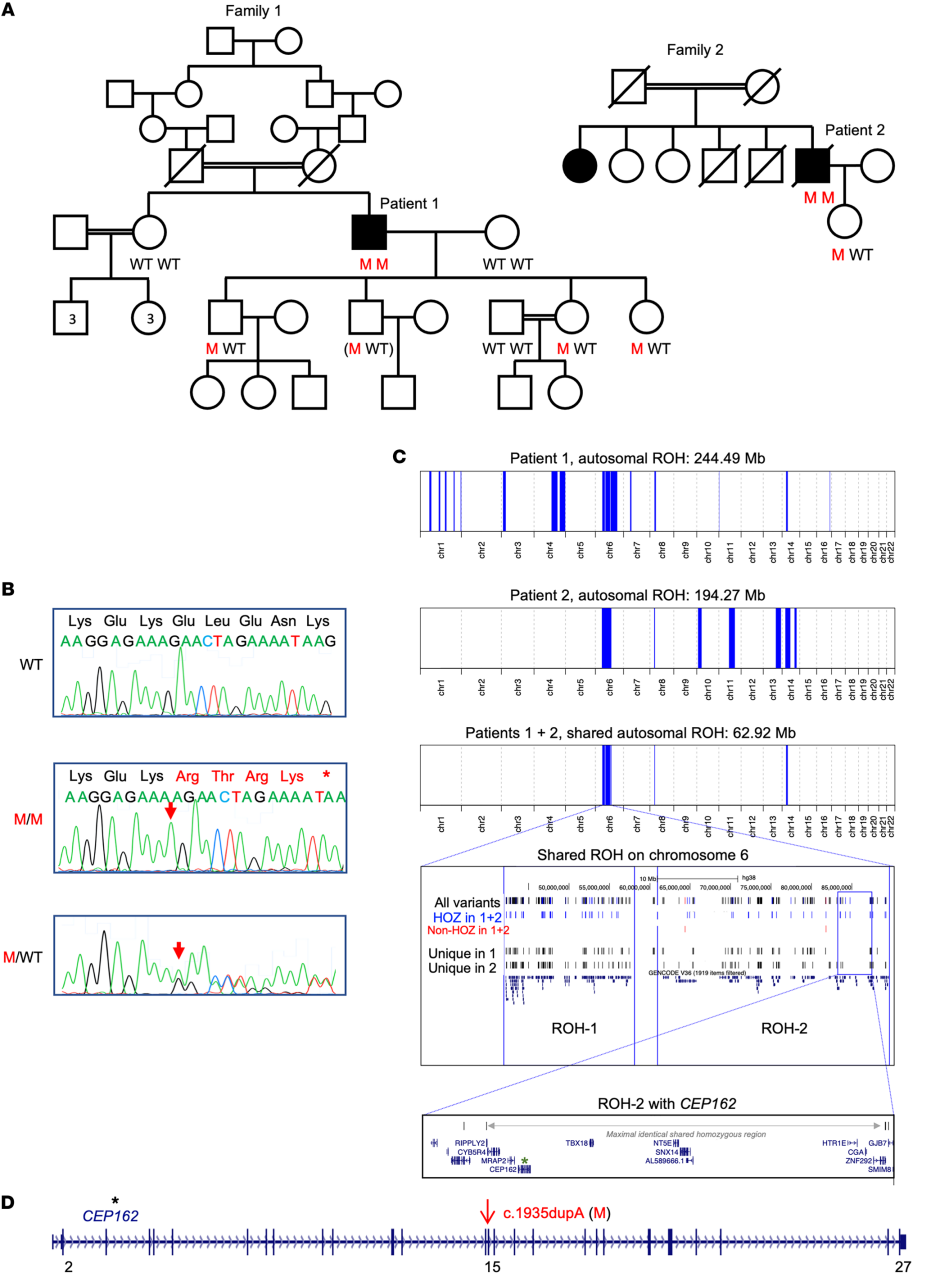
Homozygous *CEP162* frameshift variant causes RP in 2 unrelated Moroccan families. (**A**) Pedigrees with individuals who were available for genotyping of the c.1935dupA [p.(E646R*5)] in *CEP162*. M, mutation. (**B**) Electropherograms of an individual with WT sequence (upper panel), patient 1 (middle; homozygous 1-bp insertion with frameshift and premature termination codon), and a heterozygous carrier (bottom; all children of the patients). (**C**) ROH on chromosome 6, comprising *CEP162*, shared by patient 1 and patient 2. (**D**) Scheme of *CEP162* gene (to scale). Vertical bars: exons. The pathogenic variant resides in exon 15.

Patient 1 and patient 2 were both born to consanguineous parents in 2 unrelated Moroccan families (Figure 2A). The grandparents of patient 1, a 68-year-old male, are second-degree cousins. No pathogenic variant was identified in patient 1 by targeted next-generation sequencing (NGS) of 204 known IRD genes. Whole-exome sequencing (WES) generated 16.3 Gb of sequence, covering 97.9% of the target sequence more than 30 times. Among 701 rare variants, 41 were homozygous, further narrowed to 25 by applying a minor allele frequency (MAF) of less than 1%, excluding artifacts and filtering with runs of homozygosity (ROH). Twenty-four variants were disqualified from being a likely cause of RP due to one or more of the following: (a) the gene was associated with an unrelated disease; (b) there are homo- or hemizygous individuals in gnomAD; or (c) the variant was predicted as neutral or benign. A single variant remained, a 1 bp insertion in exon 15 of *CEP162*, NM_014895.3:c.1935dupA, causing a frameshift and premature nonsense codon [p.(Glu646Argfs*5), subsequently designated as E646R*5; Figure 2, B and D]. In the other annotated *CEP162* isoform, the insertion corresponds to NM_001286206.1:c.1707dup, p.(Glu570Argfs*5).

Patient 2’s older affected sister, living in North Morocco, was not available for testing. WES of the proband generated 40.6 million reads, with 99.0% of reads mapping to the target sequences, providing an average coverage of more than 30 times. Assessment of 275 RetNet genes did not reveal any likely pathogenic variants. An exome-wide analysis revealed 1,298 rare variants (MAF < 1%), 190 of which were homozygous. These were reduced to 58 variants after filtering with ROH (Figure 2C and Supplemental Table 1); and, based on the aforementioned criteria, they were further reduced to the *CEP162* c.1935dupA variant.

Segregation analysis in both families supported a pathogenic nature of the truncating *CEP162* variant (Figure 2, A and B and Supplemental Figure 2) which was absent from gnomAD (v.2.1.1). No homozygous *CEP162* loss-of-function (LoF) variants were found in gnomAD. The variant was found neither in 70 RP patients from North Africa, including 43 from Morocco, by targeted testing, nor in WES data from 1,184 cases with suspected inherited retinal diseases (IRD). The largest ROH in both patients from the 2 families was located on chromosome 6 (Figure 2C and Supplemental Table 1) and included the *CEP162*:c.1935dupA variant, putting it forward as a potential founder allele (Figure 2C).

### Mutated c.1935dupA CEP162 mRNA escapes nonsense-mediated decay in patient fibroblasts, allowing for expression of truncated CEP162 protein.

Fibroblasts from patient 1 were compared with control human dermal fibroblasts (Hdfa). Quantitative reverse-transcription PCR (qRT-PCR) analysis revealed that *CEP162* transcript levels were significantly reduced in patient fibroblasts (Supplemental Figure 3A). Anisomycin treatment was used to determine whether the patient’s *CEP162* transcript underwent nonsense-mediated decay (NMD). Increased expression of *CEP162* after anisomycin treatment was observed in both patient and control cells (Supplemental Figure 3B), suggesting that a basal level of *CEP162* transcript physiologically undergoes NMD.

To determine whether the truncated CEP162-E646R*5 protein is expressed in patient fibroblasts, we immunoblotted control and patient fibroblast lysates with an antibody that recognizes the N-terminus of CEP162 before truncation. Patient lysates only had a band at the predicted size of truncated CEP162 (at approximately 75 kDa) compared with the full-length CEP162 (at approximately 160 kDa) band in control lysates (Supplemental Figure 3C). In addition, probing with the C-terminus anti-CEP162 antibody produced a full-length CEP162 band in the control lysate, but no corresponding or truncated band in the patient lysate (Supplemental Figure 3D). We concluded that, despite the lower levels of *CEP162* mRNA, the residual transcript does not undergo complete NMD, resulting in expression of the truncated CEP162-E646R*5 protein in patient cells.

### CEP162-E646R*5 mutant protein binds microtubules but is unable to associate with centrioles or CEP290.

**Figure 3:**
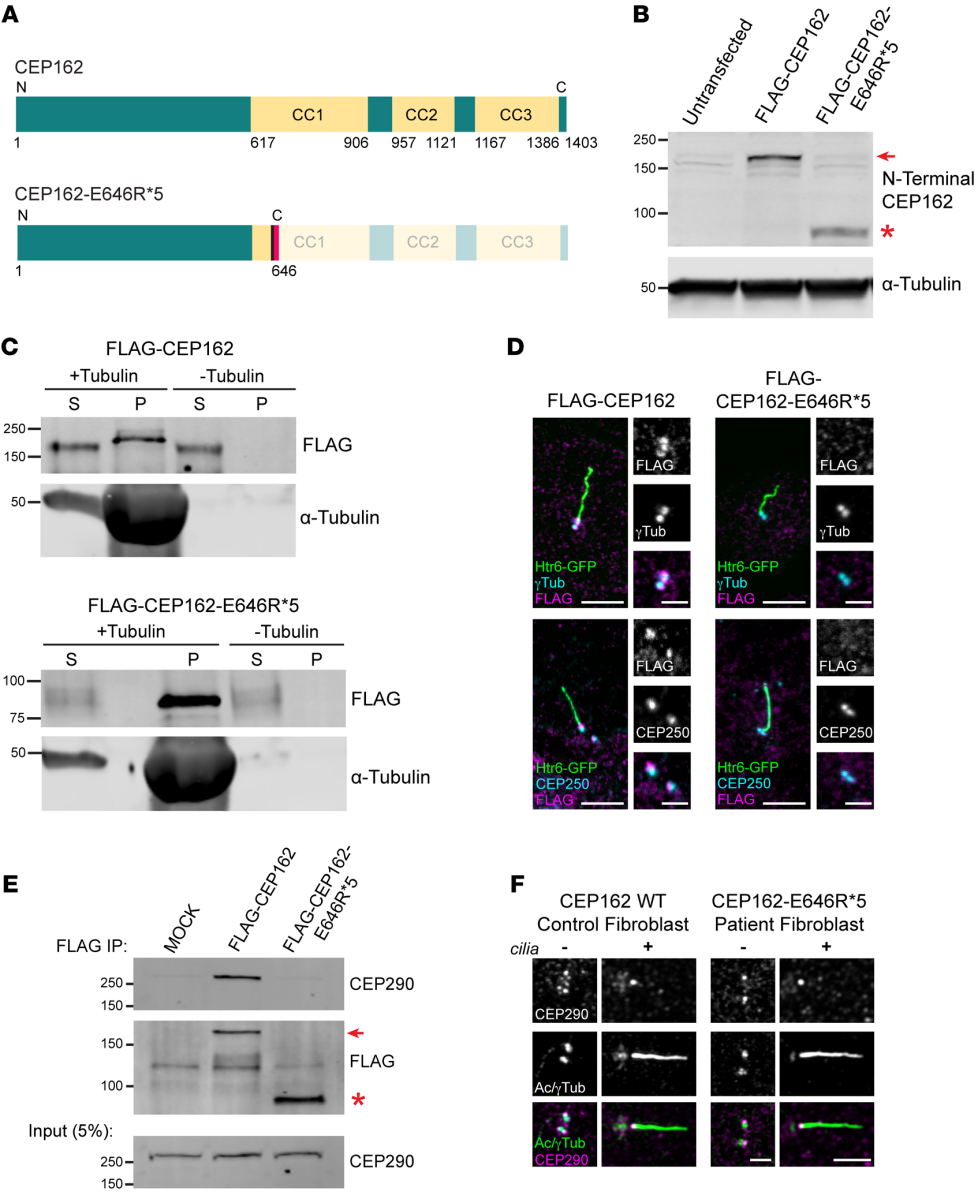
Effect of E646R*5 mutation on CEP162 protein expression and localization. (**A**) Scheme of human CEP162 protein with 3 C-terminal CC domains and the truncated CEP162-E646R*5 mutant protein. The aa residues are given for each scheme. (**B**) Western blot of 239T cell lysates from untransfected control, transfected FLAG-CEP162 (approximately 165 kDa, red arrow) and FLAG-CEP162-E646R*5 (approximately 80 kDa, red asterisk). Blots were probed for CEP162 to detect expressed protein and α-tubulin was used as a loading control. (**C**) Microtubule binding assay. Purified FLAG-CEP162 or FLAG-CEP16-E646R*5 was copelleted with taxol-stabilized microtubules. CEP162 was probed by anti-FLAG antibodies and pelleted microtubules were detected by anti–α-Tubulin antibodies. S, supernatant; P, pellet. (**D**) Serum-starved IMCD3 cells coexpressing Htr6-GFP and FLAG-CEP162 or FLAG-CEP162-E646R*5. Transfected cells were identified by GFP (green) fluorescence in the cilium. FLAG (magenta) was coimmunostained with either γ-tubulin (cyan, basal bodies) or CEP250 (cyan, proximal-end centriolar protein). Scale bars: 5 μm and 2 μm. (**E**) FLAG IP from mock, FLAG-CEP162 (approximately 165 kDa, red arrow), or FLAG-CEP162-E646R*5 (approximately 80 kDa, red asterisk) transfected 293T cells lysates. Eluates shown on top and inputs below. Blots probed with anti-CEP290 and anti-FLAG antibodies. (**F**) CEP290 (magenta) immunostaining with acetylated/γ-tubulin (Ac/γTub, green) in control and patient fibroblasts, with (+) and without (–) cilia. Scale bars: 2 μm.

Human CEP162 comprises 1,403 aa with 3 coiled-coil (CC) stretches in its C-terminus: CC1 (residues 617–906), CC2 (residues 957–1,121), and CC3 (residues 1,167–1,386). CEP162 associates with microtubules through its CC1/CC2 domains and with centrioles through its CC3 domain (10). The c.1935dupA mutation results in early truncation of the protein after the first 29 aa of the CC1 domain (Figure 3A). Truncated FLAG-CEP162-E646R*5 produced an approximately 80 kDa band compared with the full-length FLAG-CEP162 band at roughly 165 kDa (Figure 3B and Supplemental Figure 4A). We performed a microtubule binding assay to determine whether the CEP162-E646R*5 mutant protein binds microtubules. Immunoprecipitated FLAG-tagged human full-length CEP162 or E646R*5 mutant protein were copelleted with taxol-stabilized microtubules. Figure 3C shows that both full-length FLAG-CEP162 and truncated FLAG-CEP162-E646R*5 were found in the microtubule pellets, suggesting that the residual CC1 domain is sufficient to bind microtubules.

To determine whether this truncated mutant protein associated with the centrioles, we cotransfected the FLAG-tagged CEP162 constructs with the ciliary marker Htr6-GFP into IMCD3 cells and serum starved them for 24 hours to ciliate the cells (Figure 3D). Full-length FLAG-CEP162 was present at the basal bodies of Htr6-GFP-positive cilia, where it colocalized with γ-tubulin. In addition, costaining with a proximal-end centriole protein, CEP250, showed proper localization of FLAG-CEP162 to the distal end of the centrioles. In contrast, the mutant FLAG-CEP162-E646R*5 was not detected at the basal body (Figure 3D).

It was previously shown that CEP162 interacts with CEP290 through its CC1/CC2 domain (10). To determine whether the mutant CEP162-E646R*5 protein interacted with endogenous CEP290, we performed FLAG IPs from our transfected 293T cells that expressed either full-length or mutant CEP162. FLAG-CEP162-E646R*5 was unable to pull down CEP290; however, CEP290 localization at the centrioles and TZ was normal in the patient fibroblasts (Figure 3, E and F). Also, no difference in CEP290 protein levels was observed in patient fibroblasts compared with controls (Supplemental Figure 5).

### Patient fibroblasts have delayed ciliation.

**Figure 4:**
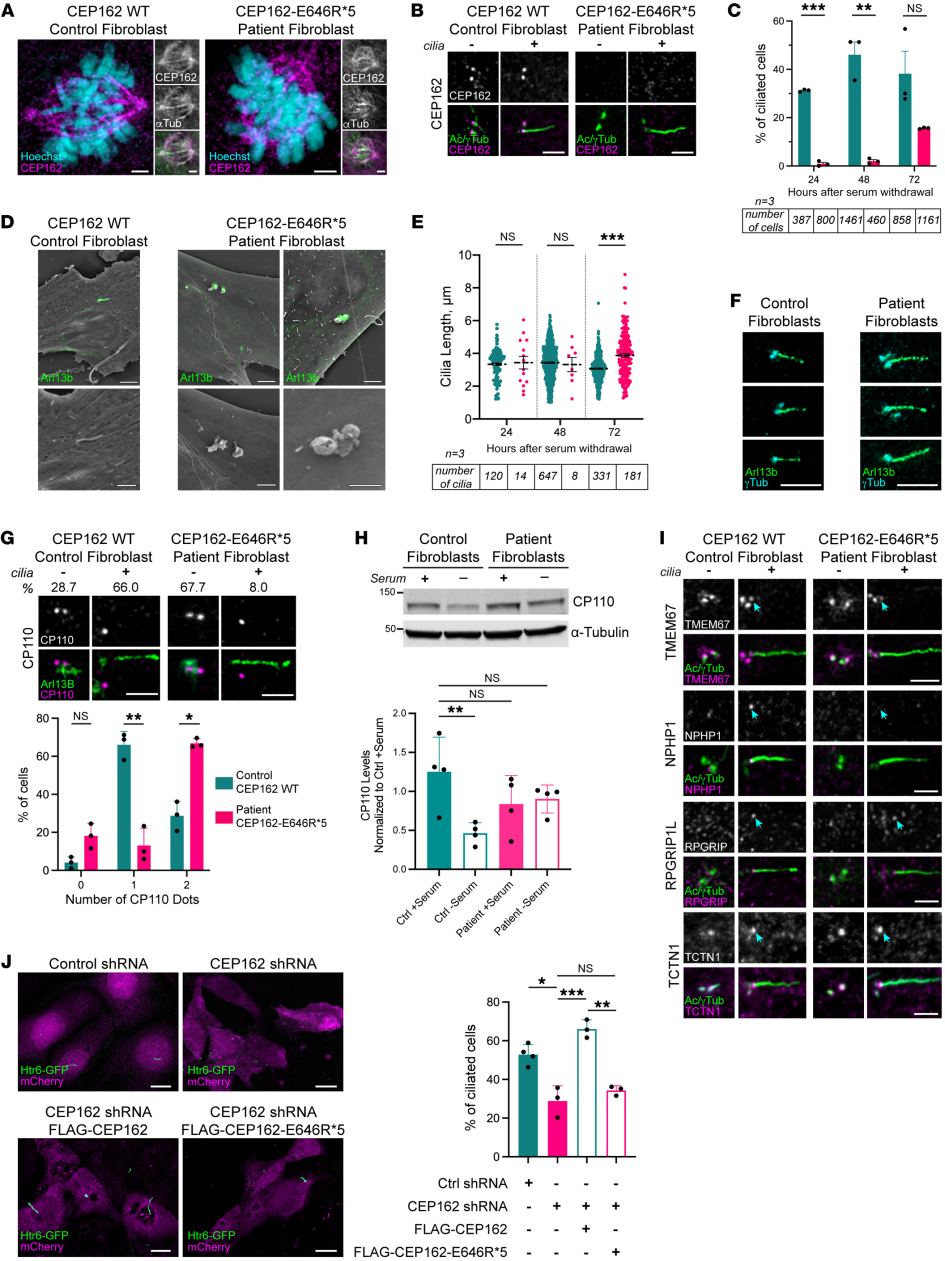
CEP162-E646R*5 localizes to the mitotic spindle in patient fibroblasts, but its absence from the basal body delays ciliation. (**A**) Control and patient fibroblasts costained with CEP162 (magenta) and α-tubulin (green, mitotic spindle). (**B**) Control and patient fibroblasts costained with CEP162 (magenta) and Ac/γTub (green). Scale bars: 2 μm. (**C**) Percent ciliation in control and patient fibroblasts 24, 48, or 72 hours after serum withdrawal. A Welch’s 2-tailed *t* test performed at each time point: ***P = 0.00012 (24 hours), **P = 0.0396 (48 hours), *P* = 0.4083 (72 hours; NS). (**D**) CLSEM performed in control and patient fibroblasts by staining cilia for Arl13b (green). Top: merged images. Bottom: magnified SEM. Scale bars: 4 μm and 2 μm. (**E**) Cilia length measurements. A Welch’s 2-tailed *t* test performed at each timepoint: *P* = 1 (24 hours; NS), *P* = 1 (48 hours; NS), ***P < 0.001 (72 hours). (**F**) Control and patient fibroblast cilia stained with Arl13b (green) and γ-tubulin (cyan). Scale bar: 5 μm. (**G**) Immunostaining of CP110 (magenta) in control and patient fibroblasts, ± cilia (Percent observed indicated above). Bar graph quantifying CP110 dots. 2-way ANOVA, *P* = 0.1701 (0 dots; NS), **P = 0.0068 (1 dot), *P = 0.0329 (2 dots). (**H**) Western blot from control and patient fibroblast lysates after 24 hours ± serum were probed for CP110 and α-tubulin. Bar graph quantifying levels of CP110 normalized to control + serum, below. Ordinary 1-way ANOVA, **P = 0.0096 (control + serum versus control – serum), *P* = 0.1929 (control + serum versus patient + serum; NS), *P* = 0.3042 (control + serum versus patient – serum; NS). (**I**) Immunostaining of TZ proteins TMEM67, RPGRIP1L, NPHP1, and TCTN1 (magenta) costained with Ac/γTub (green) in control and patient fibroblasts, ± cilia. Cyan arrows mark TZ. Scale bars: 2 μm. (**J**) Htr6-GFP cilia (green) in control or CEP162 shRNA targeted cells (mCherry, magenta) and coexpressing either FLAG-CEP162 or FLAG-CEP162-E646R*5. Scale bars: 10 μm. Bar graph shows percent ciliation in control or CEP162 shRNA expressing IMCD3 cells, right. Ordinary 1-way ANOVA, *P = 0.0012 (control versus CEP162 shRNA), ***P < 0.0001 (CEP162 versus FLAG-CEP162 shRNA), **P = 0.0002 (FLAG-CEP162 versus FLAG-CEP162-E646R*5 shRNA), *P* = 0.6186 (CEP162 versus FLAG-CEP162-E646R*5 shRNA; NS). For all graphs, error bars represent SD.

CEP162 is present at the centrioles throughout the cell cycle, and its microtubule-binding activity is believed to direct its localization to the mitotic spindle during cell division (10). Patient fibroblasts expressing the CEP162-E646R*5 truncated protein had a normal growth rate, and no aneuploidy was detected in 30 metaphases. Consistent with our result showing that microtubule binding was retained by truncated CEP162-E646R*5, CEP162 staining decorated the microtubule spindles of dividing cells in the patient fibroblasts similar to control fibroblasts (Figure 4A). This result suggests that truncated CEP162-E646R*5 localization and function was normal during fibroblast mitosis, which is expected, since CEP162’s role in postmitotic quiescence has only been described for neurons (7, 8).

As for ciliary localization, we found that CEP162 colocalizes with γ-tubulin–positive centrioles in control fibroblasts but was not detected in patient fibroblasts (Figure 4B). While control fibroblasts produced cilia within 24 hours, very few cilia in patient fibroblasts were observed before 72 hours of serum withdrawal (Figure 4C). We used correlative light and scanning-electron microscopy (CLSEM) to examine the structure of the arrested cilia in the patient fibroblasts and compared them with normal cilia produced in control fibroblasts. While control fibroblasts had normal Arl13b-positive primary cilia, patient fibroblasts only produced Arl13b-positive blebs on their surface (Figure 4D). Additionally, polyglutamylated tubulin staining was not observed in the stalled patient cilia, indicating ciliogenesis was halted prior to axoneme extension in patient fibroblasts (Supplemental Figure 4B). Together, this suggested that the ciliary membrane had fused with the plasma membrane, resulting in a bulbous bleb on the cell’s surface.

By 72 hours after serum withdrawal, the number of cilia produced in the patient fibroblasts were not significantly different from those produced in the control fibroblasts (Figure 4C). Ciliary length was measured for every cilium and imaged at the 3 time points. At 72 hours, the patient cilia were significantly longer than the control cilia (*P* < 0.001, Figure 4, E and F). Together, our data suggest that, in patient fibroblasts, ciliogenesis is paused before axoneme extension, but these cells can ultimately overcome the loss of CEP162 at the centrioles and produce cilia. Accumulation of ciliary membrane before axoneme elongation could affect the final length of the cilium in the patient fibroblasts.

### Persistence of CP110 at the mother centriole delays primary ciliogenesis in patient fibroblasts.

To determine the stage at which ciliogenesis is paused in patient fibroblasts, we analyzed the localization of molecular components involved in cilia formation. First, maturation of the basal body appeared to be normal, as proteins involved in ciliary vesicle fusion (e.g., EHD1), distal appendages formation (e.g., CEP164), and IFT machinery recruitment (e.g., IFT88) were normal (Supplemental Figure 6A). Following ciliary vesicle formation, CP110, a distal-end centriole protein that prevents microtubule nucleation, is removed from the mother centriole and degraded to enable proper axoneme elongation (11). This regulatory step was previously reported to be unaffected by the loss of CEP162 in RPE1 cells (10). In patient fibroblasts, however, quantification of CP110 dots revealed persistence of CP110 at the mother centriole of stalled cilia after 48 hours of serum withdrawal (Figure 4G). In addition, Western blot analysis for CP110 indicated that, while levels normally decrease 50% upon serum starvation in controls, levels of CP110 remained unchanged in the patient fibroblasts 24 hours after serum withdrawal (Figure 4H and Supplemental Figure 5). CP110 removal is controlled by CEP164-mediated recruitment of the serine/threonine protein kinase, TTBK2, to the distal appendages (12, 13). TTBK2 phosphorylates multiple targets, such as CEP83 and MPP9 (11, 14), that are required for CP110 removal and ciliogenesis. We found that both TTBK2 and its MPP9 substrate were localized normally in the patient fibroblasts (Supplemental Figure 6B). Although the molecules needed to remove CP110 from the mother centriole were present, this process was delayed by the loss of CEP162 at the basal body.

It was reported by Wang et al. (10) that exogenous expression of a C-terminally truncated CEP162, which maintains microtubule association but is unable to localize to centrioles, resulted in cilia that were abnormally long. In these experiments, C-terminally truncated CEP162 was found at the axoneme tip of elongated cilia where it ectopically recruited TZ components (10). Our data show that E646R*5-truncated CEP162 maintains microtubule binding during mitosis but is not localized to centrioles, suggesting it may behave similarly. To test this, we stained control and patient fibroblasts for 4 TZ proteins: TMEM67, NPHP1, RPGRIP1L, and TCTN1 (Figure 4I). We found normal localization of TCTN1, but TMEM67, NPHP1, and RPGRIP1L were not properly assembled at the ciliary base of patient fibroblasts, consistent with the complete loss of CEP162 (10). Importantly, we did not find mislocalization of any of these TZ components to the tip of the long patient cilia (Figure 4I), indicating that the CEP162-E646R*5 protein had lost its ability to recruit TZ proteins to cilia.

To confirm that CEP162-E646R*5 had no ciliary function, we performed shRNA knockdown of *CEP162* in IMCD3 cells, followed by rescue with either FLAG-tagged full-length CEP162 or CEP162-E646R*5. The shRNA was designed to target the 3′ UTR of mouse *CEP162* so that it would not interfere with overexpression rescue experiments. A single plasmid coexpressing the shRNA and soluble mCherry was used to identify transfected cells. First, we confirmed the efficiency of *CEP162* knockdown by appending the *CEP162* 3′ UTR behind the ORF of eGFP and cotransfecting AD293T cells with control or *CEP162* shRNA constructs. EGFP protein levels were reduced by approximately 75% when expressed with *CEP162* shRNA compared with the scrambled control (Supplemental Figure 7). We then used the same constructs and cotransfected with an Htr6-GFP plasmid to label the cilia in IMCD3 cells. Figure 4J shows images of the cilia in mCherry-positive cells when expressing the control or *CEP162* shRNA. *CEP162* knockdown resulted in reduced ciliation compared with control shRNA, similar to previous results (10). Expression of the FLAG-tagged full-length CEP162 rescued the loss of cilia due to *CEP162* knockdown; however, FLAG-CEP162-E646R*5 was unable to rescue the loss of cilia (Figure 4J). These results support that expression of CEP162-E646R*5 protein does not retain any ciliary function.

### CEP162 is expressed in the human retina and localizes to the basal body in mouse photoreceptors.

**Figure 5:**
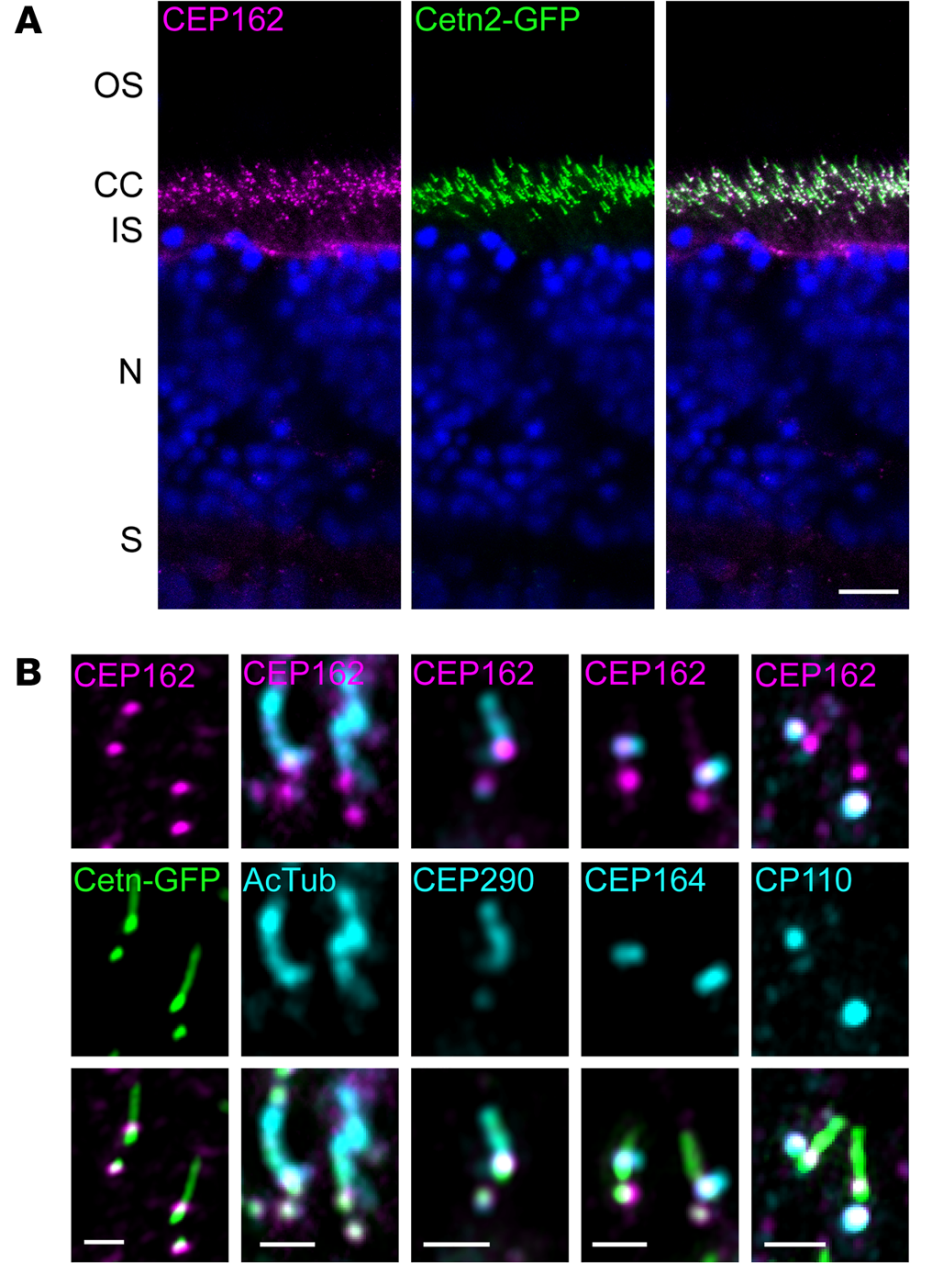
Endogenous CEP162 is localized to the distal-end of centrioles at the base of the photoreceptor outer segment in adult WT mouse retina. (**A**) Cetn2-GFP transgenic mouse retinal sections stained with an anti-CEP162 (magenta) antibody. CEP162 is localized at the photoreceptor connecting cilium, which is marked by GFP fluorescence (green). Scale bar: 10 μm. (**B**) High-resolution Airyscan images: CEP162 (magenta) staining decorates the distal ends of each centriole of the basal body at the base of the connecting cilium (Cetn2-GFP, green). Additional Airyscan images of CEP162 counterstained with multiple ciliary markers: Acetylated Tubulin (AcTub), CEP290, CEP164, and CP110 (cyan). Scale bar: 1 μm.

We examined *CEP162* expression in single-cell transcriptional data of the human neural retina (15). This revealed high expression in all retinal cell types, especially in ganglion cells (Supplemental Figure 8A). Immunostaining for CEP162 on human retinal sections showed expression throughout the human retina (Supplemental Figure 8, B–F), similar to expression previously determined in chicken retina (7). CEP162 is a centriolar protein; to determine its precise localization in photoreceptors, we used Cetn2-GFP transgenic mice that have fluorescently labelled centrioles (16). In photoreceptors, mother and daughter centrioles form 2 adjacent GFP dots and the connecting cilium emerges as a GFP streak from the mother centriole. CEP162 staining is localized to the distal end of both mother and daughter centrioles (Figure 5A). Airyscan images were acquired to determine the precise localization of CEP162 in relation to several ciliary markers, including acetylated tubulin for the axoneme, CEP290 for the connecting cilium, CEP164 for the distal appendages, and CP110 for the daughter centriole (Figure 5B). CEP162 decorated the distal ends of each centriole at the base of the photoreceptor outer segment in WT mouse retinas.

### CEP162-E646R*5 is unable to localize to the basal body in mouse photoreceptors but can rescue neuronal cell death.

**Figure 6:**
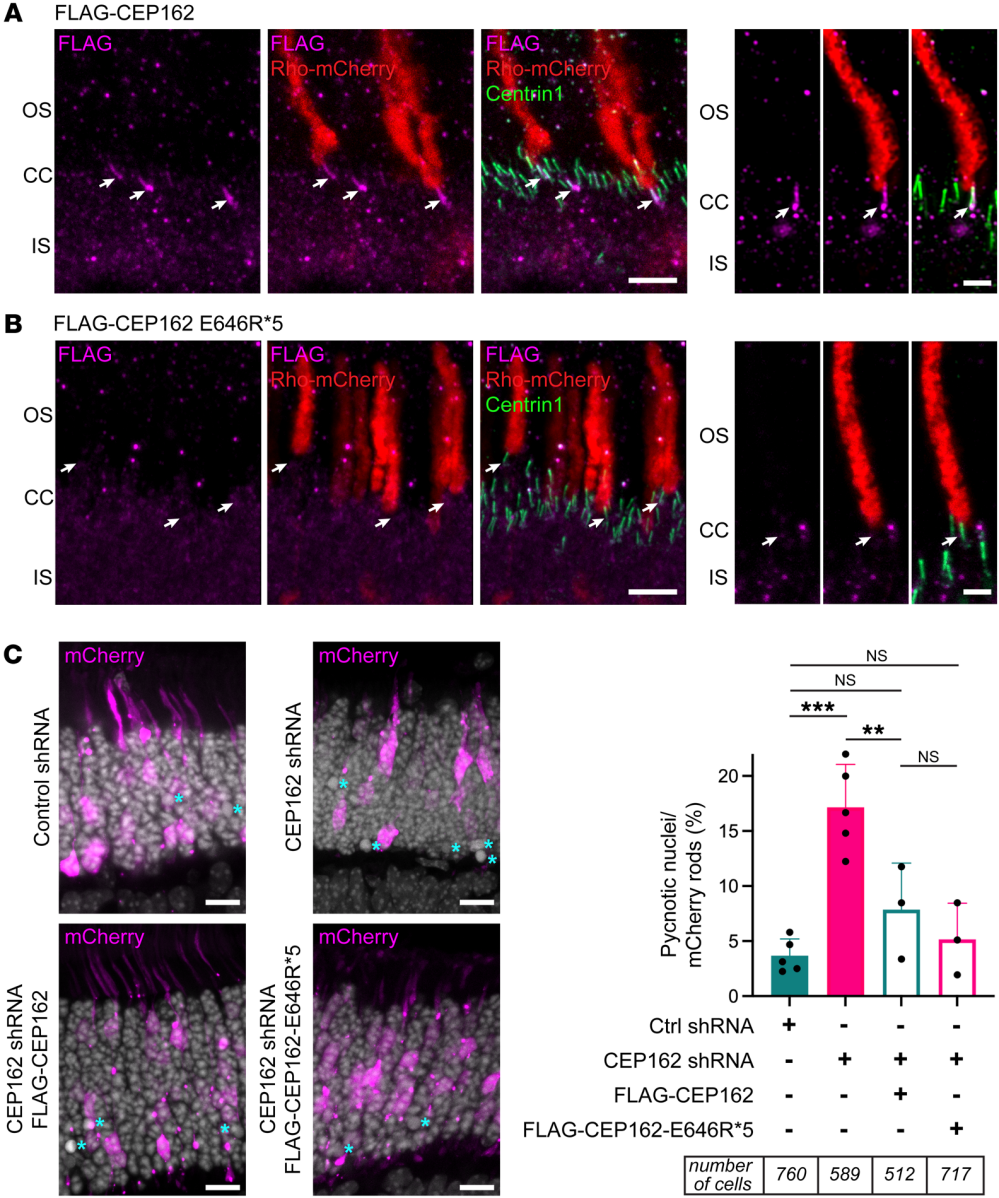
CEP162-E646R*5 mutant protein does not localize to centrioles in adult mouse rod photoreceptors but does participate in retinal neurogenesis. (**A** and **B**) Adult (P21) WT mouse retinal sections expressing either FLAG-CEP162 (**A**) or FLAG-CEP162-E646R*5 (**B**, magenta) and rhodopsin-mCherry (Rho-mCherry, red) as a transfection marker. Centrin1 (green) immunostaining was conducted to label the connecting cilium. In the transfected rods, overexpressed FLAG-CEP162 is predominantly localized to the basal body (arrows) but is also be seen within the connecting cilium. Scale bars: 5 μm and 2 μm. (**C**) Developing mouse retina (P0–P1) were electroporated with control or *CEP162* shRNA plasmids coexpressing a soluble mCherry (magenta), and *CEP162* knockdown was rescued with either FLAG-CEP162 or FLAG-CEP162-E646R*5. Retinal sections were collected at P14 for analysis. Scale bar: 5 μm. Within a mCherry-positive retinal section, cell death was assessed by counting pycnotic nuclei labeled with DAPI (grey, cyan asterisk) and normalizing to mCherry cells to account for variations in electroporation efficiency. Ordinary 1-way ANOVA, ***P = 0.0001 (control versus *CEP162* shRNA), **P = 0.01 (*CEP162* shRNA versus FLAG-CEP162), *P* = 0.3389 (control versus FLAG-CEP162; NS), *P* = 0.9217 (control versus FLAG-CEP162-E646R*5; NS), *P* = 0.746 (FLAG-CEP162 versus FLAG-CEP162-E646R*5; NS).

To determine the localization of truncated CEP162-E646R*5 mutant protein in photoreceptors, we employed in vivo electroporation (17) to express FLAG-tagged full-length and E646R*5-mutant *CEP162* constructs in mouse rods. We coexpressed Rho-mCherry to label the outer segment in transfected rods and stained retinas with anti-Centrin1 antibodies to label the connecting cilium in all photoreceptors. Figure 6A shows that FLAG-CEP162 was primarily localized to the basal body but also found within the connecting cilium in rods with Rho-mCherry-labeled outer segments. The presence of FLAG-CEP162 within the connecting cilium is likely an overexpression artifact but could also be due to epitope masking of endogenous CEP162 within the connecting cilium. In contrast, FLAG-CEP162-E646R*5-mutant staining does not localize to the basal body of Rho-mCherry-labeled outer segments (Figure 6B). This suggests that the truncated CEP162-E646R*5-mutant protein was unable to properly localize to the centrioles in photoreceptors.

Using a retroviral antisense knockdown strategy in developing chicken eyes, CEP162, or QN1, was reported to play a key role in differentiation of retinal neurons (7). We found that CEP162-E646R*5 maintains microtubule binding at the mitotic spindle in fibroblasts, so it could possibly function in neuronal differentiation. To test this, we in vivo electroporated *CEP162* shRNAs during retinal development and rescued with full-length or truncated *CEP162* constructs. Figure 6C shows representative retinal sections from P15 mice expressing a single plasmid coexpressing control or *CEP162* shRNA and soluble mCherry to identify electroporated rods. Pycnotic nuclei were counted within each electroporated area and normalized to the number of mCherry-positive rods. Knockdown of *CEP162* increased cell death approximately 4-fold compared with the control, which could be rescued by expression of either full-length or CEP162-E646R*5 (Figure 6C). Assessment of cell death is not generally used to assay retinal development, however, this result suggests that CEP162 played a critical function in the mouse developing retina and, importantly, we found that the truncated CEP162-E646R*5 retained this function.

In conclusion, our data suggest that CEP162-E646R*5 retained microtubule binding at the mitotic spindle, allowing it to function during neuroretina development, yet was absent from the ciliary basal body, limiting recruitment of a few TZ proteins, likely underlying late-onset RP in both patients.

## Discussion

Human ciliopathies are a diverse group of syndromic and nonsyndromic diseases that involve several organ systems, as cilia are ubiquitous cellular organelles (18). Understanding how ciliary genes act in a tissue-specific manner to produce the wide variety of human phenotypes remains an active area of study. Here, we identified a homozygous frameshift variant in RP patients from 2 unrelated families. We found that *CEP162* was expressed in the human retina and localized to the basal body of outer segments in mature photoreceptors. Additionally, we provide strong functional evidence that *CEP162* plays a dual role in the retina to support retinal development and maintain recruitment of key ciliary proteins.

In patient fibroblasts, low levels of *CEP162* mRNA were preserved and sufficient to produce a heavily truncated CEP162 protein. We show that the residual truncated CEP162 protein maintained microtubule binding and was present at the mitotic spindles in patient-derived fibroblasts. These cells also had a normal complement of chromosomes and growth rates, consistent with previous *CEP162* knockdown in RPE1 cells (12). CEP162’s role in cell division was previously attributed to postmitotic arrest of retinal neurons (7, 8). We found that knockdown of *CEP162* in the developing mouse retina increased neuronal cell death, which was rescued by expression not only of full-length, but also truncated CEP162. This suggests that the truncated CEP162-E646R*5 protein retains function in neuroretina development, potentially due to its preserved microtubule binding activity. Our finding that the neurogenesis function was maintained supports the diagnosis of late-onset RP in the 2 patients. Mutations that disrupt CEP162 microtubule binding would likely result in defective retinal neurogenesis in addition to ciliopathy phenotypes.

In quiescent cells, CEP162 is known to play a functional role in ciliogenesis by promoting TZ assembly (12). Truncated CEP162-E646R*5 protein was unable to localize to the ciliary basal body. The loss of CEP162 from the cilia resulted in reduced recruitment of some TZ proteins to cilia in patient fibroblasts. We confirmed through rescue experiments that the truncated CEP162 was unable to restore the loss-of-function phenotype at the cilium. In the patient fibroblasts, we found that cilia formation was delayed due to the persistence of CP110 at the mother centriole. CP110 caps the distal end of centrioles, and its removal and degradation from the mother centriole is a prerequisite for ciliation. Other aspects of early ciliogenesis, like the acquisition of distal appendages and fusion of the ciliary vesicle to the plasma membrane, were unaffected in the patient fibroblasts. Interestingly, after extended serum starvation, there was a dramatic increase in generation of abnormally long cilia, showing that ciliogenesis did occur in the absence of CEP162.

It is interesting to speculate that perhaps the retinal defect in the patients was not due to impaired ciliogenesis per se, as we found that cilia could still be formed, but instead due to reduced recruitment of TZ proteins, such as NPHP1, to the photoreceptor cilium. This could result in structural defects that are particularly detrimental to the outer segment, impairing photoreceptor maintenance rather than formation, and clinically manifesting as late-onset RP and not as congenital retinal disease, as in *CEP290*-associated Leber congenital amaurosis (19).

In summary, our data uncover a dual role for the centriolar protein, CEP162, in retinal neurons to ensure proper neurogenesis and ciliary TZ assembly. Our genomic, cell-based, and in vivo data show that, although the mitotic function of CEP162 during neuronal development is maintained, specific loss of CEP162 function at the cilium resulted in a novel retinal ciliopathy in humans. These findings highlight the tissue-specific roles of ciliary proteins and may be instrumental for future studies exploring diverse ciliopathies.

## Methods

### Clinical assessment

Patients underwent ophthalmologic evaluation, including slit lamp examination, widefield color fundus photography, and blue light autofluorescence (CLARUS 500; Carl Zeiss Meditec Inc.), as well as spectral domain optical coherence tomography (Spectralis SDOCT; Heidelberg Engineering, and PLEX Elite 9000; Carl Zeiss Meditec Inc.). An abdominal ultrasound — and additional CT in patient 2 — was conducted to exclude cysts of the kidneys and liver fibrosis.

### Genetic and genomic studies

#### Genomic testing.

For the index patients of both families, Patients 1 and 2, genomic DNA (gDNA) was extracted from leukocytes and pooled libraries were paired-end sequenced on an Illumina NextSeq500 (Illumina). Nucleotides were numbered with nucleotide A of the ATG as c.1 (HGVS guidelines; http://www.hgvs.org). Maximum MAF cut off was 1%. Variants were checked for presence in ClinVar and HGMD databases (20, 21) and classified based on ACMG and ACGS guidelines (22–25). For patient 1, targeted NGS (IDT xGen Inherited Diseases Panel v1.0; IDT Integrated Technologies), WES (IDT xGen Exome Research Panel v1.0; IDT Integrated Technologies), and variant assessment were conducted as reported previously (26). After targeted NGS, variant analysis was performed for 204 genes known to associate with IRD, including *RPGR*-*ORF15* (SeqNext module of SeqPilot software; JSI Medical Systems). Genes associated with the Human Phenotype Ontology (HPO) term “Retinal dystrophy” (HP:0000556) were searched for pathogenic homozygous or potentially compound-heterozygous variants (varSEAK Pilot 2.0.2; JSI Medical Systems). After WES, the CCG pipeline (27), and interface (Varbank 2.0; https://varbank.ccg.uni-koeln.de/varbank2/) were used for data analysis as described (28, 29). ExAC and gnomAD databases (30) (as of February 2021) were searched for candidate variants identified in the homozygous state. In patient 2, WES was performed using the SureSelect XT Human All Exon V6 kits (Agilent). CLC Genomics Workbench version 7.0.5 (CLCBio) was used for read mapping against the human genome reference sequence (NCBI, GRCh37/hg19), and removal of duplicate reads, coverage analysis, and quality-based variant calling, followed by further variant annotation using Alamut Batch were performed (Interactive Biosoftware). Variants were scored as heterozygous or homozygous and assessed with our in–house variant filtering and visualization tool. There were 275 RetNet genes assessed (version 4 of the in-house RetNet panel), followed by assessment of homozygous variants in the exome. Copy number variations (CNVs) were assessed using ExomeDepth (v1.1.10) (31).

#### Segregation analysis and screening for the CEP162 variant c.1935dupA p.(E646R*5) in patients with IRD.

Available members of families 1 and 2 and 70 patients with RP from North Africa, including 43 from Morocco, were tested for the *CEP162* variant c.1935dupA p.(E646R*5) by Sanger sequencing (primer sequences available in Supplemental Table 1). Pseudonymized WES data from 1,184 IRD cases were also analyzed for presence of the variant (Ghent University Hospital).

#### ROH detection and haplotype analysis.

In patient 1, ROH were identified using HomozygosityMapper and applying default settings (32). ROH of less than 3 kb adjacent to each other were merged manually, reducing ROH from 71 to 67. In patient 2*,* ROH were initially mapped by genome–wide single-nucleotide polymorphism (SNP) chip analysis (HumanCytoSNP-12 BeadChip platform; Illumina). ROH (> 1 Mb) were identified using PLINK software (33) integrated in ViVar (34) and ranked according to length and number of consecutive homozygous SNPs. For both patients 1 and 2, ROH were determined with AutoMap (35) using VCF files from both patients (hg38). After identification of individual ROH, shared ROH (based on coordinates) were determined (Figure 2C and Supplemental Table 1). To define the shared ROH on chromosome 6 and the common haplotype containing the *CEP162* variant, all WES variants on chromosome 6 were considered, irrespective of their zygosity (Figure 2C).

#### Fibroblast culture from skin biopsy.

Fibroblasts were isolated from a dermal punch biopsy of the upper arm of patient 1 as described (36). The skin biopsy was cut into 18–24 equally sized pieces and plated on a 6-well plate coated with 0.1% Gelatin. Fibroblasts migrated out of the skin biopsies after 7–10 days and were split on 2 75 cm^2^ flasks after 3–4 weeks. At 90% confluency, the 2 flasks were split into 3 separate 175 cm^2^ flasks. After isolation, fibroblasts were routinely cultured in IMDM/Glutamax containing 15% FBS and 1% penicillin/streptomycin (all from Invitrogen). Normal Primary Hdfa (ATCC; PCS-201-012) served as controls.

#### Karyotyping.

We analyzed 30 metaphases from cultured patient 1 fibroblasts using standard procedures.

### Mouse studies

Albino Cetn2-GFP transgenic mice were obtained from Jackson Labs (Strain 027967). The *Pde6b^Rd1^* mutation was removed by backcrossing to a BALB/cJ albino mouse from Jackson Labs. Albino CD-1 WT mice were from Charles River (Strain 022). All mice were housed under a 12-hour light/12-hour dark cycle. Experimenters were not blinded to genotype.

For in vivo electroporation, retinal transfection of CD-1 neonatal mice was conducted as previously described (17, 37, 38). In P0–P2 neonates, 2 μg/μl shRNA and 1 μg/μl rhodopsin-mCherry plasmid DNA was deposited subretinally. Retinal tissue was collected at P20–P22 for protein localization studies and collected at P14–P15 for nuclear counts after shRNA knockdown.

### In vitro and in vivo functional studies

DNA constructs were generated using standard PCR-based subcloning methods. *Homo sapiens* centrosomal protein 162 (CEP162), transcript variant 1, mRNA (NM_014895.3) was obtained from GeneCoepia (HOC20483). A FLAG-tag was added by overlap extension PCR to the N-terminus. All DNA constructs were cloned between a 5′ *Age*I and a 3′ *Not*I site, using standard T4 DNA ligation methods, and the sequence was confirmed. For mouse in vivo electroporation, the pRho plasmid was used (Addgene; 11156). For mammalian cell culture, the pEGFP-N1 vector was used (Clontech; PT3027-5A). Mutagenesis was performed using the QuikChange II XL kit (Stratagene). Cloning of the Rho-mCherry construct was previously described in Finkelstein et al. (39). For primers, see Supplemental Table 2.

#### Plasmid expression in cell culture.

AD293T or IMCD3 cells were transfected at 90%–95% confluence with DNA constructs mentioned above, using Lipofectamine 3000 transfection reagent (Invitrogen). The next day, cells were incubated in complete medium or serum-free medium for another 24 hours before analysis.

### Immunofluorescence

Supplemental Table 3 contains a complete list of antibodies and dilutions. We thank Greg Pazour (University of Massachusetts, Chan Medical School, Worcester, Massachusetts, USA) and Bryan Tsou (Sloan Kettering Institute, New York, New York, USA) for the gift of antibodies.

#### Cetn2-GFP mouse retinal cross-sections.

Immunostaining was carried out as described (40). Briefly, fresh eyecups were embedded in OCT compound and snap frozen in methylbutane cooled with liquid nitrogen. Ten μm–thick retinal cryosections were blocked in 5% donkey serum and 0.05% Triton X-100 in PBS for 15 minutes before incubated in primary antibodies diluted in block for 1 hour. Sections were rinsed, fixed for 5 minutes in 1% paraformaldehyde in PBS, rinsed and incubated with secondary antibodies in block for 2 hours at 22°C.

#### Electroporated mouse retinal cross-sections.

The immunostaining protocol was adapted from Robichaux, et al. (41). Electroporated retinas with mCherry expression were dissected in supplemented mouse ringers, pH 7.4 and approximately 313–320 mOsM. Fresh retinas were blocked in 10% normal donkey serum, 0.3% saponin, 1 × cOmplete Protease (Millipore Sigma) diluted in supplemented mouse ringers for 2 hours at 4°C. Primary antibodies diluted in block were incubated for 20–22 hours, rinsed and incubated in secondary antibodies for 2 hours, all at 4°C. Retinas were rinsed, fixed in 4% paraformaldehyde for 30 minutes at 22°C, embedded in 4% agarose (Thermo Fisher Scientific), after which 100 μm vibratome retinal-sections were collected.

#### Cultured cells.

Between 50 and 60 × 10^3^ fibroblasts were plated onto 13 mm poly L-lysine glass coverslips (Corning) in DMEM-F12 culture media containing 10% FBS and grown overnight in a 37°C incubator with 5% CO_2_. The next day, serum-free media was added for 24, 48, or 72 hours to induce ciliation. Cells were placed on ice for 10 minutes before fixation in 1% paraformaldehyde for 5 minutes followed by 15 minutes in ice-cold methanol. Cells were then rinsed, permeabilized for 5 minutes with 0.1% SDS in PBS, rinsed, and blocked with 0.1% Triton-X, 5% normal donkey serum, and 5% BSA in PBS. Primary antibodies diluted in block were incubated overnight at 4°C, rinsed, and incubated with secondary antibodies for 1 hour at 22°C.

For all conditions, 10 μg/ml Hoechst 33342 was added with donkey secondary antibodies conjugated to Alexa Fluor 488, 568, or 647 (Jackson ImmunoResearch). Coverslips (no. 1.5, Electron Microscopy Sciences) were mounted with Prolong Glass (Thermo Fisher Scientific). Images were acquired using a Zeiss Observer 7 inverted microscope with a 63 × oil-immersion objective (1.40 NA) and a LSM 800 confocal with Airyscan detector controlled by Zen 5.0 (Carl Zeiss Microscopy). Image manipulation was limited to adjusting the brightness level, image size, rotation, and cropping using FIJI (ImageJ).

### IP

A 10 cm plate of AD293T cells was transfected using Polyethyleneimine (PEI; Sigma-Aldrich) at a 6:1 ratio of PEI-to-DNA with 10 μg of mock, FLAG-CEP162, or FLAG-CEP162-E646R*5 construct. After 24 hours, cells were lysed in 1% NP40 in PBS, cleared by centrifugation at 21,000*g* for 20 minutes at 10°C and the supernatant was incubated on anti-FLAG M2 magnetic beads (Sigma-Aldrich) rotating overnight at 4°C. Immunoprecipitants were eluted with 100 mM 3 × FLAG peptide (Sigma-Aldrich) in lysis buffer rotating for 1 hour at 4°C and processed for immunoblotting. See complete unedited blots in Supplemental Figure 9.

### Microtubule pelleting assays

FLAG-CEP162 or FLAG-CEP162-E646R*5 immunoprecipitated in BRB80 (80 mM PIPES, 1 mM MgCl_2_, 1 mM EGTA, ph 6.8, diluted from a 5 × stock with 1 × protease inhibitor) from 5 separate 10 cm plates of AD293T cells was added to taxol-stabilize microtubules. The binding reaction was added on top of a 1 mL cushion (40% glycerol in BRB80 with 10 μM taxol, 1 × protease inhibitor and 1 mM GTP) and spun in fixed-angle rotor TLA120.1 at 100,000*g* for 40 minutes at 25°C. Soluble and pellet fractions were collected, run on SDS-PAGE, and Western blotted for FLAG.

### Statistics

All data represent at least 3 independent experiments. The data are presented as a mean ± SD or SEM as indicated in the figure legends. 2-tailed Welch’s *t* tests were performed for direct comparisons between patient and control fibroblasts. In case of multiple comparisons, *P* values were adjusted using the Bonferroni method. Ordinary 1-way ANOVAs were used for multiple comparison of CP110 levels in control and patient ± serum conditions, qRT-PCR comparisons, and shRNA knockdown plus rescue experiments. A 2-way ANOVA was used for comparing the number of CP110 dots between control and patient fibroblasts. All statistical analyses were performed using Prism9 (GraphPad). *P*_adj_ <0.05 was considered significant for all statistical tests performed. The employed statistical tests, along with the resulting *P*_adj_ values, are listed in each figure legend.

### Study approval

All individuals involved gave their informed consent prior to inclusion in this study. All investigations were conducted according to the Declaration of Helsinki, and the study was approved by the Ethics Committee of the University Hospital of Cologne, Ghent (EC UZG 2017/1540) and Brussels (A420701EI13L). Mice protocols were approved by IACUC at the University of Michigan (registry number A3114-01).

## Author contributions

UZ, JNP, EDB, and HJB were responsible for conception of the project. NN, UZ, JNP, EDB, and HJB participated in project design. NN, KVS, SL, MB, ADR, SK, MJ, JRW, JW, HMT, NG, MVH, JW, RM, SGD, JVD, AH, HS, LM, AFR, TL, KD, DR, KMW, MVL, HR, FH, PN, HT, UZ, JNP, EDB, and HJB contributed to acquisition, analysis, and interpretation of the data. NN, KVS, MB, ADR, UZ, JNP, EDB, and HJB assisted in drafting the manuscript. NN, KVS, JRW, HMT, MVH, UZ, JNP, EDB, and HJB revised the manuscript.

## Supplementary Material

Supplemental data

## Version 1 03/02/2023

In-Press Preview

## Version 2 04/17/2023

Electronic publication
