# Supplementary material for: CEP162 deficiency causes human retinal degeneration and reveals a dual role in ciliogenesis and neurogenesis
Source: J Clin Invest. 2023 Apr 17;133(8):e161156. doi: 10.1172/JCI161156 (PMC10104899; doi:10.1172/JCI161156)
Supplement: Supplemental data [file jci-133-161156-s177.pdf]

Supplemental Data

CEP162 deficiency causes human retinal degeneration and reveals a dual role in ciliogenesis and neurogenesis

Nafisa Nuzhat[1], Kristof Van Schil[2,3], Sandra Liakopoulos[4,5], Miriam Bauwens[2,3], Alfredo Dueñas Rey[2,3], Stephan Käseberg[6], Melanie Jäger[7,8], Jason R. Willer[9], Jennifer Winter[6], Hanh M. Truong[10], Nuria Gruartmoner[2,3], Mattias Van Heetvelde[2,3], Joachim Wolf[11], Robert Merget[11], Sabine Grasshoff-Derr[12], Jo Van Dorpe[13,14], Anne Hoorens[13,14], Heidi Stöhr[15], Luke Mansard[16], Anne-Françoise Roux[16], Thomas Langmann[17], Katharina Dannhausen[17], David Rosenkranz[18], Karl Martin Wissing[19], Michel Van Lint[20], Heidi Rossmann[21], Friederike Häuser[21], Peter Nürnberg[22,23], Holger Thiele[22], Ulrich Zechner[6,18], Jillian N. Pearring[1,9,10]*, Elfride De Baere[2,3]*, Hanno J. Bolz[18,24]*

[1]Department of Cell and Developmental Biology, University of Michigan, Ann Arbor, Michigan, USA. [2]Department of Biomolecular Medicine, Ghent University, Ghent, Belgium. [3]Center for Medical Genetics, Ghent University Hospital, Ghent, Belgium. [4]Cologne Image Reading Center, Department of Ophthalmology, University Hospital of Cologne, Cologne, Germany. [5]Department of Ophthalmology, Goethe University, Frankfurt, Germany. [6]Institute of Human Genetics, University Medical Center Mainz, Mainz, Germany. [7]Department of Ophthalmology, Justus-Liebig-University Giessen, Giessen, Germany. [8]Augenarztpraxis Bad Brückenau, Bad Brückenau, Germany. [9]Department of Ophthalmology and [10]Cell and Molecular Biology Program, University of Michigan, Ann Arbor, Michigan, USA. [11]Department of Radiology and [12]Department of Pediatric Surgery, Bürgerhospital, Frankfurt am Main, Germany. [13]Department of Diagnostic Sciences, Ghent University, Ghent, Belgium. [14]Department of Pathology, Ghent University Hospital, Ghent, Belgium. [15]Institute of Human Genetics, University of Regensburg, Regensburg, Germany. [16]Laboratoire de Génétique Moléculaire, CHU de Montpellier, Université de Montpellier, Montpellier, France. [17]Laboratory for Experimental Immunology of the Eye, Department of Ophthalmology, Faculty of Medicine and University Hospital Cologne, Cologne, Germany. [18]Senckenberg Centre for Human Genetics, Frankfurt am Main, Germany. [19]Department of Nephrology and [20]Department of Ophthalmology, Brussels University Hospital, Jette, Belgium. [21]Institute of Clinical Chemistry and Laboratory Medicine, University Medical Center Mainz, Mainz, Germany. [22]Cologne Center for Genomics (CCG), University of Cologne, Faculty of Medicine and University Hospital Cologne, Cologne, Germany. [23]Center for Molecular Medicine Cologne, University of Cologne, Cologne, Germany. [24]Institute of Human Genetics, University Hospital of Cologne, Cologne, Germany.
*These authors contributed equally to this work.

Correspondence to:
Hanno J. Bolz, MD. Present address: Bioscientia Human Genetics, Institute for Medical Diagnostics, Konrad-Adenauer-Str. 17, 55218 Ingelheim, Germany; Institute of Human Genetics, University Hospital of Cologne, Kerpener Str. 34, Cologne, Germany. Phone: +49 6132781206. Email: hanno.bolz@icloud.com

Elfride De Baere, MD, PhD. Center for Medical Genetics Ghent (CMGG), Ghent University Hospital; Department of Biomolecular Medicine, Ghent University; Ghent University Hospital, Corneel Heymanslaan 10, B-9000 Ghent, Belgium. Email: Elfride.DeBaere@UGent.be

Jillian Pearring, PhD. Department of Ophthalmology, Department of Cell and Developmental Biology, University of Michigan, Ann Arbor, MI, U.S.A. Email: pearring@umich.edu

SUPPLEMENTAL TABLE 1

AutoMap output for Patient 1 and Patient 2.

chr	pos	ref	alt	patient 1	patient 2	compare
chr6	42194650	A	G	ALT/ALT	ALT/ALT	IDENTICAL
chr6	42208382	G	T	ALT/ALT	ALT/ALT	IDENTICAL
chr6	42632581	A	G	ALT/ALT	ALT/ALT	IDENTICAL
chr6	42745880	A	G	ALT/ALT	ALT/ALT	IDENTICAL
chr6	43046560	T	C	ALT/ALT	ALT/ALT	IDENTICAL
chr6	43338608	A	G	ALT/ALT	ALT/ALT	IDENTICAL
chr6	43369033	C	T	ALT/ALT	ALT/ALT	IDENTICAL
chr6	44113981	G	A	ALT/ALT	ALT/ALT	IDENTICAL
chr6	44275822	A	G	ALT/ALT	ALT/ALT	IDENTICAL
chr6	44280214	T	G	ALT/ALT	ALT/ALT	IDENTICAL
chr6	44282428	A	G	ALT/ALT	ALT/ALT	IDENTICAL
chr6	44286012	A	G	ALT/ALT	ALT/ALT	IDENTICAL
chr6	44287722	G	A	ALT/ALT	ALT/ALT	IDENTICAL
chr6	44300634	T	C	ALT/ALT	ALT/ALT	IDENTICAL
chr6	44301456	C	T	ALT/ALT	ALT/ALT	IDENTICAL
chr6	44307274	T	C	ALT/ALT	ALT/ALT	IDENTICAL
chr6	44313075	C	T	ALT/ALT	ALT/ALT	IDENTICAL
chr6	44352811	T	A	ALT/ALT	ALT/ALT	IDENTICAL
chr6	46596080	A	T	ALT/ALT	ALT/ALT	IDENTICAL
chr6	46642168	C	T	ALT/ALT	ALT/ALT	IDENTICAL
chr6	46655961	T	C	ALT/ALT	ALT/ALT	IDENTICAL
chr6	46689320	A	G	ALT/ALT	ALT/ALT	IDENTICAL
chr6	46691168	C	G	ALT/ALT	ALT/ALT	IDENTICAL
chr6	46705206	A	G	ALT/ALT	ALT/ALT	IDENTICAL
chr6	46716485	C	T	ALT/ALT	ALT/ALT	IDENTICAL
chr6	46825438	G	A	ALT/ALT	ALT/ALT	IDENTICAL
chr6	46833334	G	T	ALT/ALT	ALT/ALT	IDENTICAL
chr6	46888363	G	A	ALT/ALT	ALT/ALT	IDENTICAL
chr6	47009541	G	A	ALT/ALT	ALT/ALT	IDENTICAL
chr6	47595956	C	T	ALT/ALT	ALT/ALT	IDENTICAL
chr6	47679106	A	G	ALT/ALT	ALT/ALT	IDENTICAL
chr6	47681486	T	G	ALT/ALT	ALT/ALT	IDENTICAL
chr6	47681837	C	T	ALT/ALT	ALT/ALT	IDENTICAL
chr6	47681958	A	G	ALT/ALT	ALT/ALT	IDENTICAL
chr6	47687006	A	T	ALT/ALT	ALT/ALT	IDENTICAL
chr6	47710719	CT	C	ALT/ALT	ALT/ALT	IDENTICAL
chr6	47714868	A	C	ALT/ALT	ALT/ALT	IDENTICAL
chr6	47716855	G	A	ALT/ALT	ALT/ALT	IDENTICAL
chr6	47879443	A	G	ALT/ALT	ALT/ALT	IDENTICAL
chr6	47879665	A	G	ALT/ALT	ALT/ALT	IDENTICAL

SUPPLEMENTAL TABLE 1 (continued)

chr6	49492265	A	G	ALT/ALT	ALT/ALT	IDENTICAL
chr6	49512037	T	C	ALT/ALT	ALT/ALT	IDENTICAL
chr6	49512062	A	T	ALT/ALT	ALT/ALT	IDENTICAL
chr6	49551201	C	G	ALT/ALT	ALT/ALT	IDENTICAL
chr6	51619163	T	C	ALT/ALT	ALT/ALT	IDENTICAL
chr6	51627086	T	C	ALT/ALT	ALT/ALT	IDENTICAL
chr6	51721973	G	A	ALT/ALT	ALT/ALT	IDENTICAL
chr6	52010452	A	C	ALT/ALT	ALT/ALT	IDENTICAL
chr6	52059976	A	G	ALT/ALT	ALT/ALT	IDENTICAL
chr6	52509521	T	TAC	ALT/ALT	ALT/ALT	IDENTICAL
chr6	52752933	C	G	ALT/ALT	ALT/ALT	IDENTICAL
chr6	52836345	C	T	ALT/ALT	ALT/ALT	IDENTICAL
chr6	53652078	T	G	ALT/ALT	ALT/ALT	IDENTICAL
chr6	53654264	A	G	ALT/ALT	ALT/ALT	IDENTICAL
chr6	53654807	C	T	ALT/ALT	ALT/ALT	IDENTICAL
chr6	54137433	A	C	ALT/ALT	ALT/ALT	IDENTICAL
chr6	55277539	A	G	ALT/ALT	ALT/ALT	IDENTICAL
chr6	55874755	A	G	ALT/ALT	ALT/ALT	IDENTICAL
chr6	56060164	A	G	ALT/ALT	ALT/ALT	IDENTICAL
chr6	64307084	TGA	T	REF/ALT	ALT/ALT	DIFFERENT
chr6	64626250	G	A	ALT/ALT	ALT/ALT	IDENTICAL
chr6	64912570	A	G	ALT/ALT	ALT/ALT	IDENTICAL
chr6	65495052	G	A	ALT/ALT	ALT/ALT	IDENTICAL
chr6	70068486	G	C	ALT/ALT	ALT/ALT	IDENTICAL
chr6	70102231	A	G	ALT/ALT	ALT/ALT	IDENTICAL
chr6	70274733	A	G	ALT/ALT	ALT/ALT	IDENTICAL
chr6	72179769	A	G	ALT/ALT	ALT/ALT	IDENTICAL
chr6	72182554	A	G	ALT/ALT	ALT/ALT	IDENTICAL
chr6	73425293	C	T	ALT/ALT	ALT/ALT	IDENTICAL
chr6	73451780	A	G	ALT/ALT	ALT/ALT	IDENTICAL
chr6	73452078	G	T	ALT/ALT	ALT/ALT	IDENTICAL
chr6	75087586	C	T	ALT/ALT	ALT/ALT	IDENTICAL
chr6	75132006	A	T	ALT/ALT	ALT/ALT	IDENTICAL
chr6	75659338	A	G	ALT/ALT	ALT/ALT	IDENTICAL
chr6	77462224	C	T	ALT/ALT	ALT/ALT	IDENTICAL
chr6	78998283	A	C	ALT/ALT	ALT/ALT	IDENTICAL
chr6	80014595	A	G	ALT/ALT	ALT/ALT	IDENTICAL
chr6	81751803	A	G	ALT/ALT	ALT/ALT	IDENTICAL
chr6	81752010	A	ACCGCCGAAGTCGCCG	REF/ALT	ALT/ALT	DIFFERENT
chr6	83524112	G	A	ALT/ALT	ALT/ALT	IDENTICAL
chr6	83591278	A	G	ALT/ALT	ALT/ALT	IDENTICAL
chr6	83593623	T	C	ALT/ALT	ALT/ALT	IDENTICAL
chr6	84174816	C	CT	ALT/ALT	ALT/ALT	IDENTICAL

SUPPLEMENTAL TABLE 1 (continued)

chr6	84764040	C	T	ALT/ALT	ALT/ALT	IDENTICAL
chr6	87260583	C	G	ALT/ALT	ALT/ALT	IDENTICAL
chr6	87284854	G	A	ALT/ALT	ALT/ALT	IDENTICAL
chr6	87284854	G	C	ALT/ALT	REF/REF	DIFFERENT
chr6	87472986	G	C	ALT/ALT	ALT/ALT	IDENTICAL
chr6	87606023	TA	T	ALT/ALT	ALT/ALT	IDENTICAL
chr6	89105111	A	G	ALT/ALT	ALT/ALT	IDENTICAL
chr6	89198154	G	A	ALT/ALT	ALT/ALT	IDENTICAL

SUPPLEMENTAL TABLE 2

List of primers used for PCR amplification and Sanger sequencing of exon 15 of *CEP162*, for qRT-PCR experiments and for the generation and sequencing verification of FLAG-tagged CEP162 plasmids.

**Primer Name**	**Sequence (5' -> 3')**	**Usage**
CEP162-E15-F1	AAATCCCACAGAAACTGATTCC	PCR and sequencing CEP162 exon 15 (Patient 1). Product size: 619 bp
CEP162-E15-R1	AGGAACCAAGGATGACAATG	PCR and sequencing CEP162 exon 15 (Patient 1). Product size: 619 bp
CEP162-E15-F2	TTAACCACCTGTGTTCAAATCTT	PCR and sequencing CEP162 exon 15 (Patient 2). Product Size: 361 bp
CEP162-E15-R2	GCAGGGAATTCAGCTTTTT	PCR and sequencing CEP162 exon 15 (Patient 2). Product Size: 361 bp
FLAG-hCEP162-F	GATTACAAGGATGACGATGACAAGGCTA ACTGTTCCCAAGAAGAG	Add N-terminal FLAG tag
Age-FLAG-F	CCCGGGATCCACCGGTGCCACCATGGA TTACAAGGATGACGA	Adding 5’ AgeI site
hCEP162-Seq-R1	CGATTTAGTTCAGCTACTTTGGAA	Sequencing human CEP162
hCEP162-Seq-R2	TTTAGCATGAGCCTGTTCCA	Sequencing human CEP162
hCEP162-Seq-R3	GCCGAAGTGCATCTTTATCC	Sequencing human CEP162
hCEP162-Seq-R4	AGGATCAGCTGCTTCTCCAA	Sequencing human CEP162
hCEP162-Seq-R5	GCCTGAGCTCCTAACTGACG	Sequencing human CEP162
hCEP162-Seq-R6	TCTCCCAATGAATGGGCTAT	Sequencing human CEP162
hCEP162-Seq-R7	AGTTCTGCGTTGGCTTGATT	Sequencing human CEP162
hCEP162-Seq-F1	CCATGAGAAGGGTCAAGGAA	Sequencing human CEP162
hCEP162-Seq-F2	AGCCTGCAGCTTTGGATAAA	Sequencing human CEP162
NotI-hCEP162-R	AGGAGTGCGGCCGCGGATCCTTAATAC TCTGGTGCATTC	Adding 3’ NotI site
hCEP162-1935dup-F	CATTCTCAGAAAAGGAGAAAAGAACTAG AAAATAAGTTGG	Insert 1935A duplication
hCEP162-1935dup-R	CCAACTTATTTTCTAGTTCTTTTCTCCTTT TCTGAGAATG	Insert 1935A duplication
hCEP162-exon5-F	AATCAAGCCAACGCAGAACT	qRT-PCR exon 5 of CEP162
hCEP162-exon6-R	ACGGTGCACCAACATACTCA	qRT-PCR exon 5 of CEP162
hCEP162-exon13-F	AAATCCCACAGAAACTGATTCC	qRT-PCR exon 14 of CEP162
hCEP162-exon14-R	GCACCCCTCCATTTATCCTC	qRT-PCR exon 14 of CEP162
hCEP162-exon25-F	TGAAACATTTCGTGGGCTTA	qRT-PCR exon 25 of CEP162
hCEP162-exon26-R	TGTGCCAGTCTTTTCCATTTT	qRT-PCR exon 25 of CEP162
hHSP90-Ex5-F	TGGAGGAACGAAGAATAAAGGA	qRT-PCR housekeeping
hHSP90-Ex6-R	TTCAGCCTCATCATCGCTTA	qRT-PCR housekeeping

hGAPDH-Ex7-F	ACACCCACTCCTCCACCT	qRT-PCR housekeeping
hGAPDH-Ex8-R	TGCTGTAGCCAAATTCGTTG	qRT-PCR housekeeping
CEP162 N-terminal-F	GGGATCAGTGGTTTTGATTTACA	CEP162 NMD Analysis
CEP162 N-terminal-R	ATATTTGGGTTCATCTTCAGGG	CEP162 NMD Analysis
CEP162 center-F	ACAGTGGTATGCTGAAAATCAGG	CEP162 NMD Analysis
CEP162 center-R	GCGTATCTTCTGCCGAATAGATG	CEP162 NMD Analysis
CEP162 C-terminal-F	CCTTTGCCAAAATGCAGTAGA	CEP162 NMD Analysis
CEP162 C-terminal-R	CTTGTTTACTCTGCAGCTCATTAA	CEP162 NMD Analysis
SRSF2 variant 3-F	GGCGTGTATTGGAGCAGATGTA	Positive Control for NMD Analysis
SRSF2 variant 3-R	CTGCTACACAACTGCGCCTTTT	Positive Control for NMD Analysis
SRSF2 variant 1-F	CGTGCCTGAAACTGAAACCA	Negative Control for NMD Analysis
SRSF2 variant 1-R	TTGCCAACTGAGGCAAAGC	Negative Control for NMD Analysis
shRNA-SCRAMBLE-F	CCGGAATGCCTACGTTAAGCTATACCTCGAGGTATAGCTTAACGTAGGCATTTTTTTG	Control shRNA
shRNA-SCRAMBLE-R	AATTCAAAAAAATGCCTACGTTAAGCTATACCTCGAGGTATAGCTTAACGTAGGCATT	Control shRNA
shRNA-CEP162-T3-F	CCGGACTTAGGACAAGGGCTATATTCTCGAGAATATAGCCCTTGTCCTAAGTTTTTG	CEP162 shRNA
shRNA-CEP162-T3-R	AATTCAAAAAACTTAGGACAAGGGCTATATTCTCGAGAATATAGCCCTTGTCCTAAGT	CEP162 shRNA
mCEP162-3UTR-F	CGGAACTTGGATCTGAGTGACC	Clone mouse CEP162 3'UTR into pEGFPN1
mCEP162-3UTR-R	ATTACTGCCTTGCCTGGACA	Clone mouse CEP162 3'UTR into pEGFPN1

SUPPLEMENTAL TABLE 3

List of antibodies used in this study including the source, catalog number, and dilution used in either immunofluorescence (IF) or Western blot (WB) experiments.

Name	Source	Catalog #	Dilution for IF	Dilution for WB
Rabbit anti-CP110	Sigma Aldrich	MABT1354	1:500	1:1000
Rabbit anti-CEP162 (N terminus)	Sigma Aldrich	HPA030170	N/A	1:500
Rat anti-CEP162	Meng-Fu Bryan Tsou, Sloan Kettering Institute	N/A	1:2000	N/A
Rabbit anti-CEP162 (C terminus)	Sigma Aldrich	HPA030173	N/A	1:1000
Rabbit anti-CEP290	Bethyl Laboratories	A301-659A	1:250	1:2000
Mouse anti-Gamma Tubulin	Sigma Aldrich	T5326	1:2000	N/A
Mouse anti-Alpha Tubulin	Sigma Aldrich	T9026	1:2000	1:8000
Mouse anti-Acetylated Tubulin	Sigma Aldrich	T7451	1:2000	N/A
Mouse anti-Polyglutamylated Tubulin B3	Sigma Aldrich	T9822	1:500	N/A
Rabbit anti-CEP250	Proteintech	14498-1-AP	1:500	N/A
Rabbit anti-FLAG	Pierce	PAS-21401	1:1000, 1 μg/retina	N/A
Mouse anti-FLAG, M2	Sigma Aldrich	F1804	1:2000	N/A
Rabbit anti-FLAG	Sigma Aldrich	F7425	N/A	1:1000
Mouse anti-Beta Actin	Santa Cruz	SC-47778	N/A	1:8000
Rabbit anti-Arl13b	Proteintech	17711-1-AP	1:2000	N/A
Mouse anti-CEP164	Novus Biologicals	NBP2-43631	1:500	N/A
Rabbit anti-RP2	Sigma Aldrich	HPA000234	N/A	1:2000
Rabbit anti-IFT88	Proteintech	13967-1-AP	1:1000	N/A
Rabbit anti-MKS3/TMEM67	Proteintech	13975-1-AP	1:500	N/A
Rabbit anti-IFT20	Greg Pazour, UMass Chan Medical School	N/A	1:100	N/A
Mouse anti-Centrin1, 20H5	Millipore	04-1624	1 μg/retina	N/A
Rabbit anti-NPHP1	Greg Pazour, UMass Chan Medical School	N/A	1:250	N/A
Rabbit anti-RPGRIP1L	Sigma Aldrich	HPA039405	1:250	N/A
Rabbit anti-EHD1	Novus Biologicals	NBP2-56412	1:500	N/A
Mouse anti-Rab8	BD Biosciences	610844	1:500	N/A
Rabbit anti-TTBK2	Sigma Aldrich	HPA018113	1:1000	N/A
Rabbit anti-MPP9	Sigma Aldrich	HPA037485	1:500	N/A
Goat anti-mCherry	Sicgen	AB0040	N/A	1:5000
Mouse anti-Living Colors (JL-8, GFP)	TaKaRa	632381	N/A	1:5000

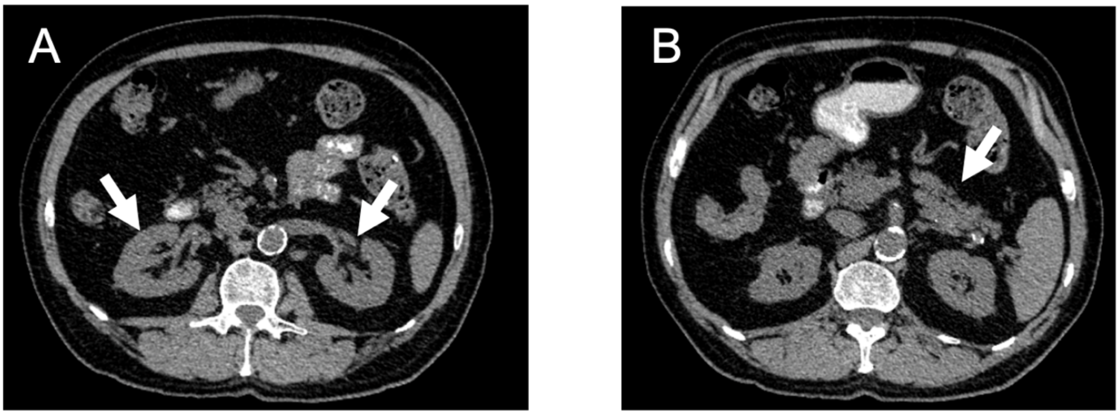

SUPPLEMENTAL FIGURE 1

Abdominal CT of Patient 2. A Kidneys (white arrows) without cysts or other morphological abnormalities. **B** Pancreas (grey arrow) with a lipomatous aspect.

A

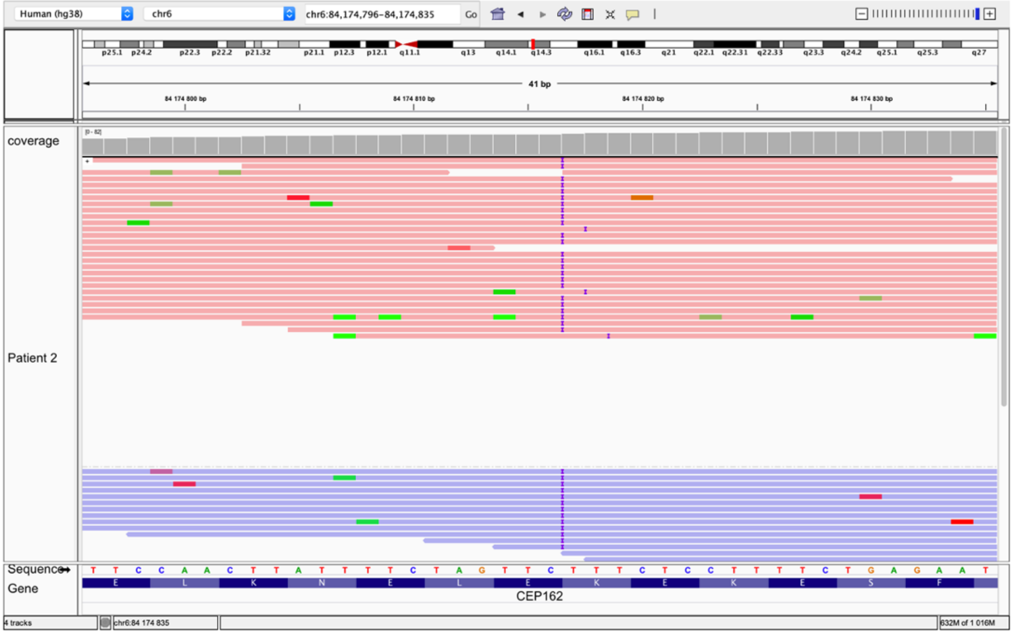

B

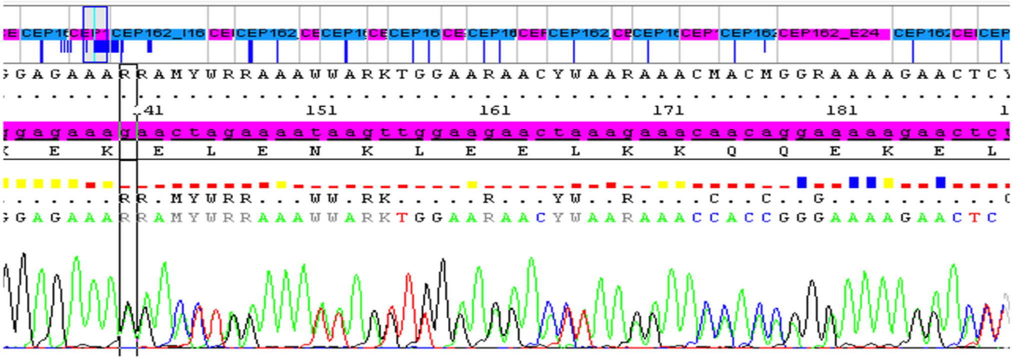

SUPPLEMENTAL FIGURE 2

Homozygous *CEP162* frameshift variant in patient 2 and segregation analysis in his daughter. A Whole-exome sequencing reads visualized in the Integrative Genomics Viewer IGV, showing the homozygous *CEP162* variant c.1935dupA [p.(E646R*5)]. **B** Sanger sequencing electropherogram of the heterozygous *CEP162* variant in the daughter of patient 2.

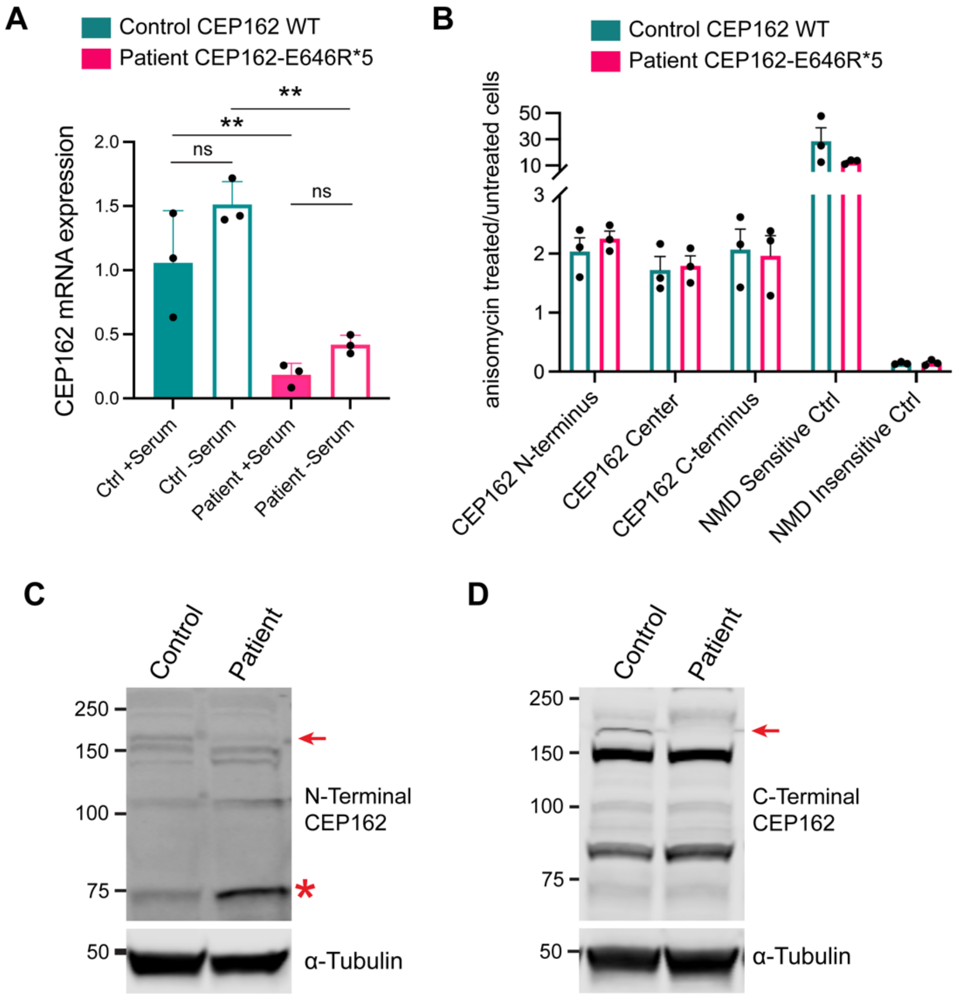

SUPPLEMENTAL FIGURE 3

***CEP162* mRNA expression is reduced, but truncated CEP162-E646R*5 protein is expressed in patient fibroblasts. A** Transcript levels of *CEP162* in control and patient fibroblasts after 24 hours +/– serum conditions. Quantitative RT-PCR using primer sets across exons 5, 14 and 25 of the *CEP162* gene was performed in triplicate from three biological replicates at each condition. We found the Ct value between each primer set was consistent providing high confidence that the entire *CEP162* mRNA was reverse transcribed. Therefore, the relative mRNA expression level in each sample was calculated by taking an average of the Ct values for all primer sets normalized to the housekeeping gene *HSP90*. Error bars represent SD. An ordinary 1-way ANOVA was performed, $P = 0.1553$ (control + serum versus control – serum; NS), $P = 0.6743$ (patient + serum versus patient – serum; NS), $^{**}P = 0.0065$ (control + serum versus patient + serum), $^{**}P = 0.0016$ (control – serum versus patient – serum). **B** Inhibition of NMD by anisomycin treatment results in a similar increase in fibroblasts of patient and controls. Bar and scatter plot of three independent experiments analyzed in duplicates by ddPCR. **C-D** Representative Western blot of control and patient fibroblast lysates. Blots were probed with an N-terminal anti-CEP162 antibody to detect full length (red arrow) and truncated (red asterisk) bands (**C**). C-terminal anti-CEP162 antibody only detected full length CEP162 (red arrow) in the control fibroblast lysates (**D**). α-tubulin was used as a loading control.

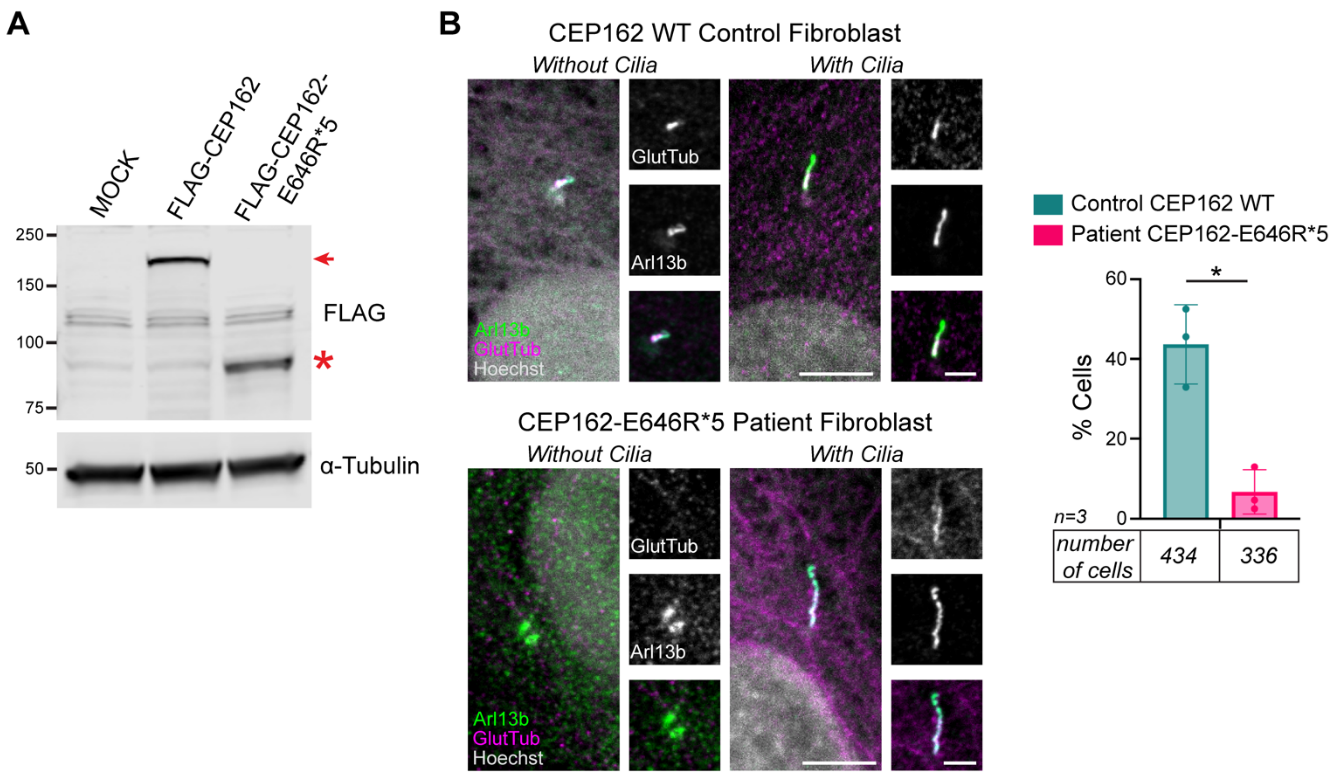

SUPPLEMENTAL FIGURE 4

FLAG-CEP162 and FLAG-CEP162-E646R*5 expression in 293T cells and glutamylated tubulin staining in control and patient fibroblasts. A Representative Western blot of 239T cell lysates from untransfected control, FLAG-CEP162 (~165 kDa, red arrow) and FLAG-CEP162-E646R*5 (~80 kDa, red asterisk). Blots were probed for anti-FLAG antibody to detect expressed protein as well as anti-α-tubulin antibody as a loading control. **B** Control and patient fibroblasts without and with a cilium were co-stained with Arl13b (green) and glutamylated tubulin B3 (GlutTub, magenta). Nuclei labeled with Hoechst 33342 (grey). Scale bars: 5 μm and 2 μm. Bar graph quantifying the % of control or patient fibroblasts with positive glutamylated tubulin staining shown on the right. A Welch's 2-tailed *t* test was performed, *P = 0.011. Error bars represent SD.

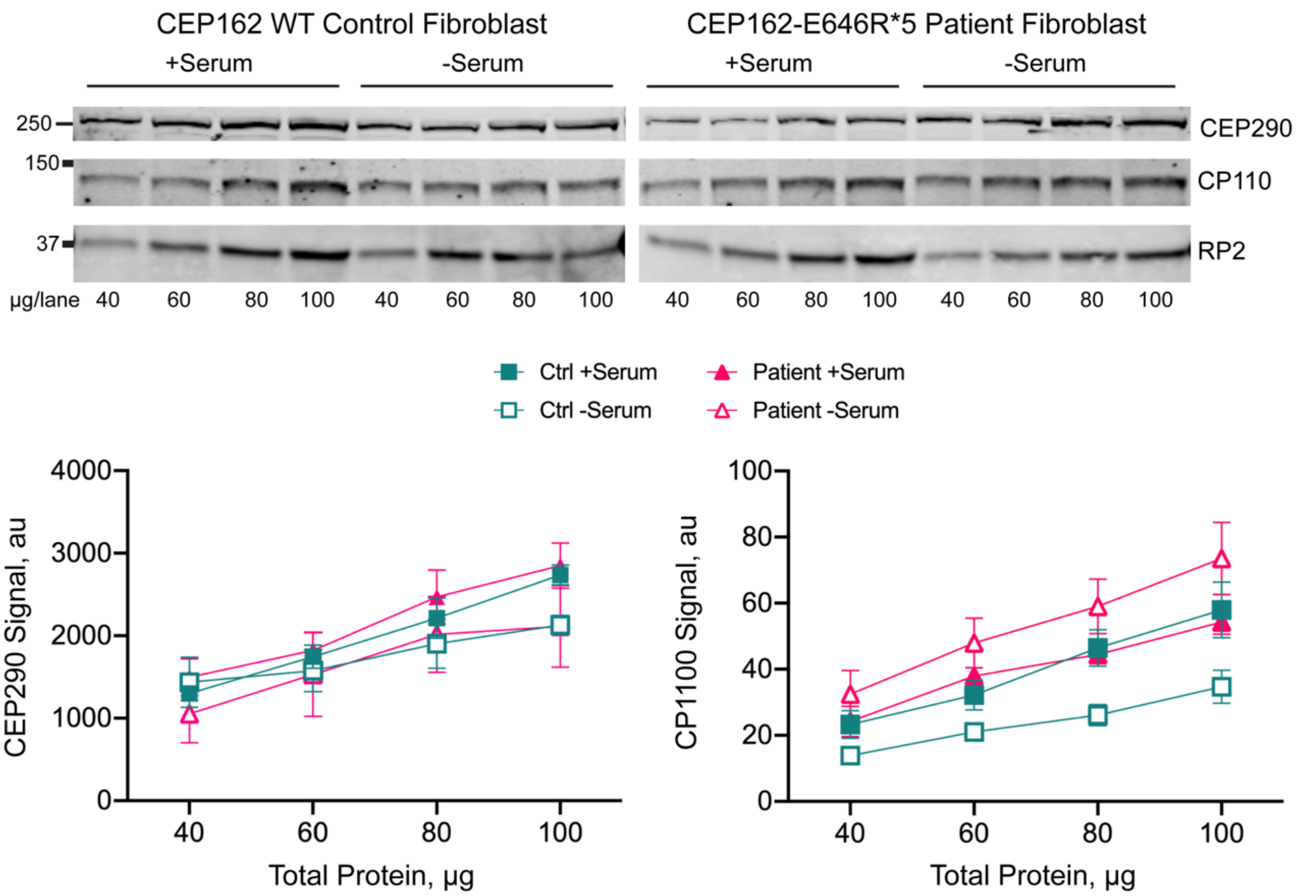

SUPPLEMENTAL FIGURE 5

Quantification of CEP290, CP110 and RP2 protein levels in CEP162-mutant patient fibroblasts compared to controls. Representative Western blots show serial dilutions of control (CEP162-WT) fibroblast and patient (CEP162-E646R*5) fibroblast lysates for CEP290, CP110 and RP2 proteins. Amount of protein lysate (µg) loaded in each lane is displayed below. The fluorescent signal produced by the CEP290 or CP110 bands in three separate experiments was plotted versus total protein loaded. The slope of the curves was used to calculate the amount of each protein in control and patient fibroblasts.

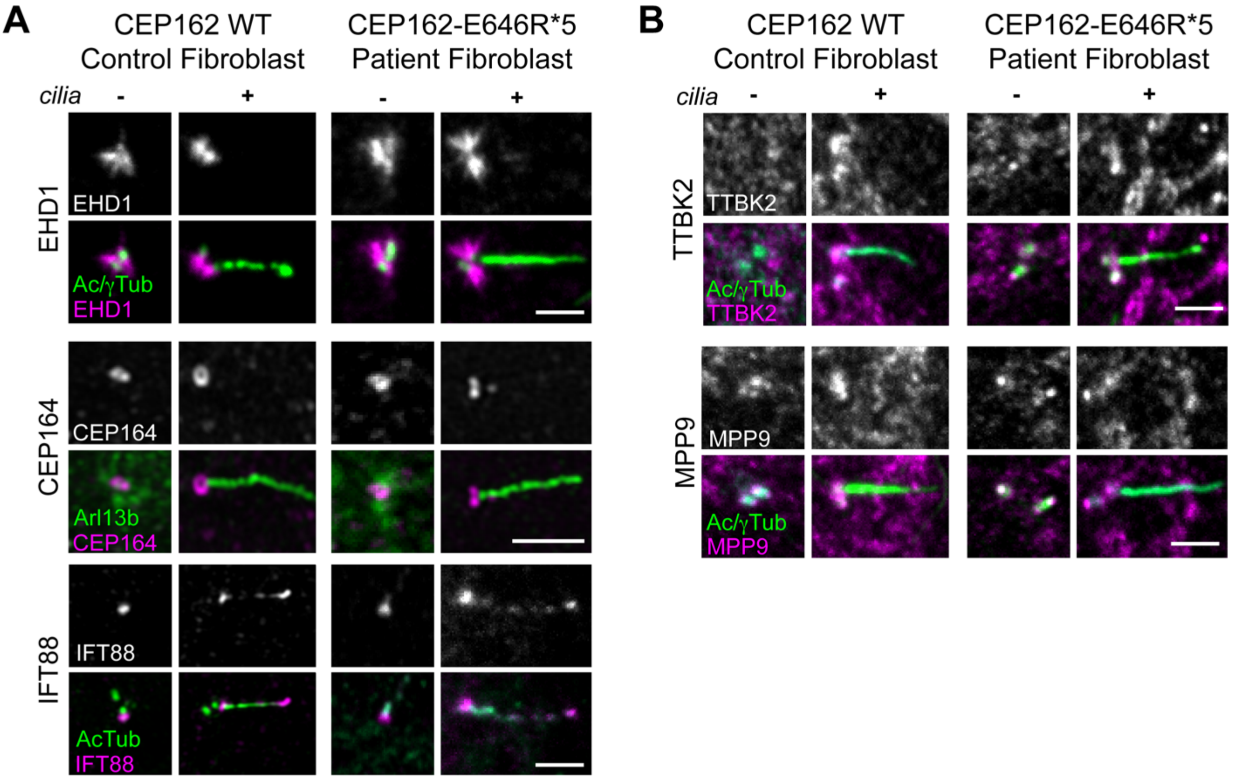

SUPPLEMENTAL FIGURE 6

Immunostaining for proteins implicated in early stages of ciliogenesis and removal of CP110 in control and patient fibroblasts. A Immunostaining of early ciliogenesis markers in control and patient fibroblasts: EDH1, CEP164, and IFT88 (magenta). **B** Molecules implicated in the removal of CP110: TTBK2 and MPP9 (magenta) are normally localized at the basal body of patient fibroblasts.

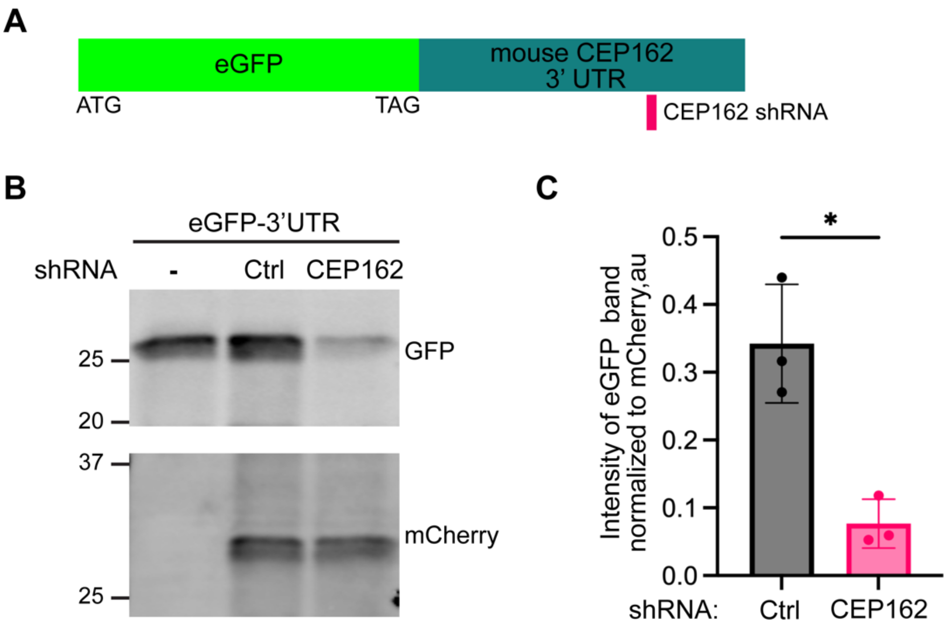

SUPPLEMENTAL FIGURE 7

Validation of mouse *Cep162* 3' UTR shRNA. A Diagram showing eGFP fused with the 3' UTR from mouse *Cep162*. The site targeted by the *Cep162* shRNA is shown below. **B** Representative Western blot of AD293 cell lysates co-expressing eGFP-3'UTR and no shRNA construct (–), scramble control shRNA (Ctrl), or CEP162 shRNA. Blot probed with anti-GFP to monitor reduction in protein expression and anti-mCherry for normalization to transfected cells. **C** Bar graph shows eGFP-3'UTR levels are reduced when co-expressed with *Cep162* shRNA. The eGFP level is normalized to mCherry to account for variations in transfection efficiency. A Welch's 2-tailed t test was performed, $*P = 0.0217$. Error bars represent SD.

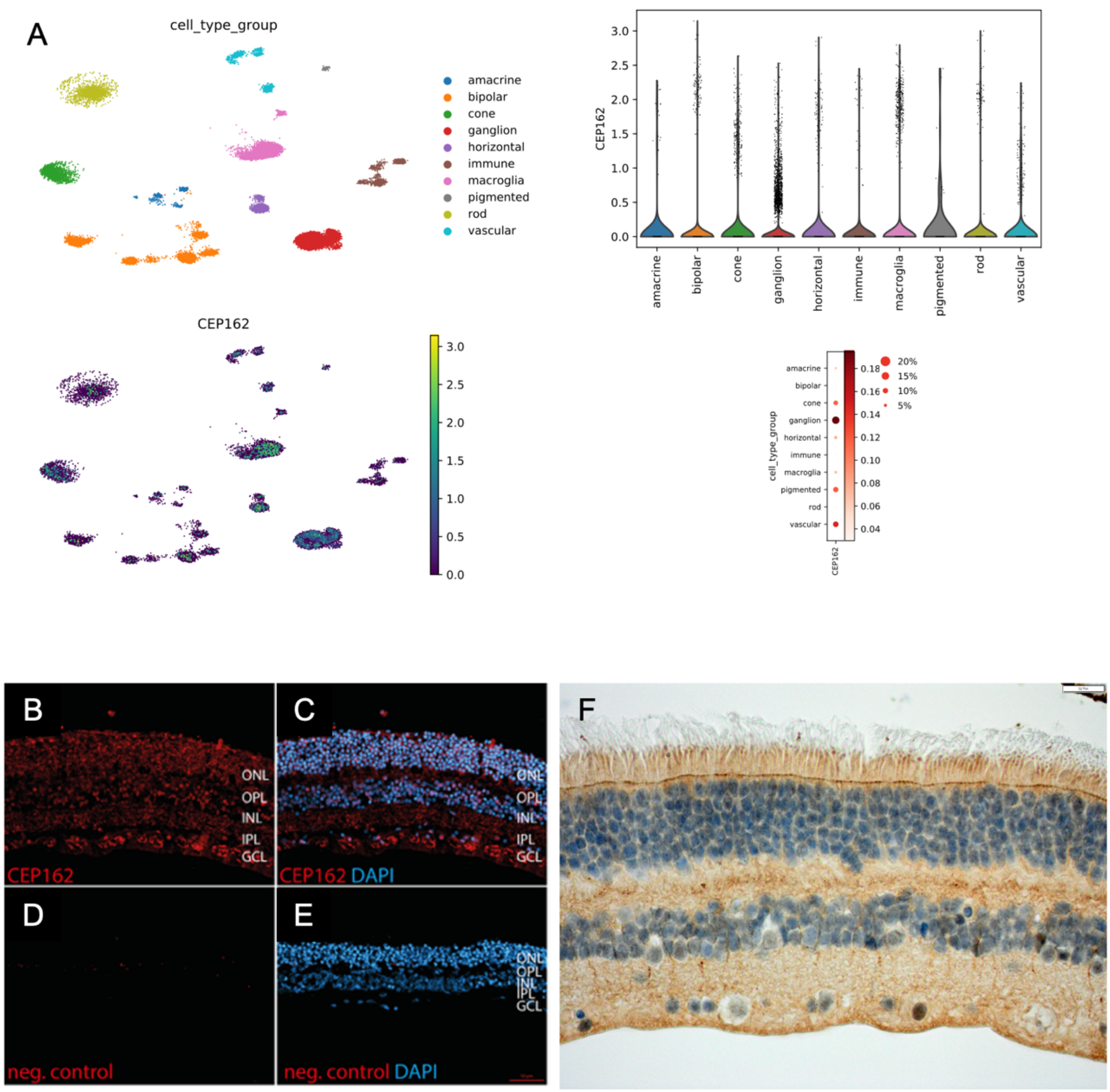

SUPPLEMENTAL FIGURE 8

Expression analysis of CEP162. A Mining of single-cell transcriptional retinal data displaying the highest expression in cells belonging to the ganglion cell population. (Left) UMAP plot of 19,768 human neural retinal cells colored based on cell type. Visualization of scaled and corrected expression on UMAP plot for *CEP162*. (Top right) Violin plot for visualization of scaled and corrected expression of *CEP162* in the 9,768 human neural retinal cells. (Bottom right) Individual dots are colored and sized to visualize both the proportion of cells in each population expressing a given gene (dot diameter size) and the average expression level of that gene (dot color intensity) in the detected cells. **B-F** CEP162 immunostaining on human retinal sections. Representative fluorescence images of human paraffin-embedded sections stained with anti-CEP162 antibody (red) (**B-C**) or negative control (**D-E**). Sections were counter-stained with DAPI (blue), displayed in **C** and **E**. Scale bars represent 100 mm. **F** Immunostaining of the CEP162 protein on human retina is visible in light brown, displaying retinal expression. Light microscopy at 400x. Scale bars represent 100 mm. Abbreviations: GCL, ganglion cell layer; INL, inner nuclear layer; IPL, inner plexiform layer; OPL, outer plexiform layer; ONL, outer nuclear layer; OLM, outer limiting membrane; PR, photoreceptors.

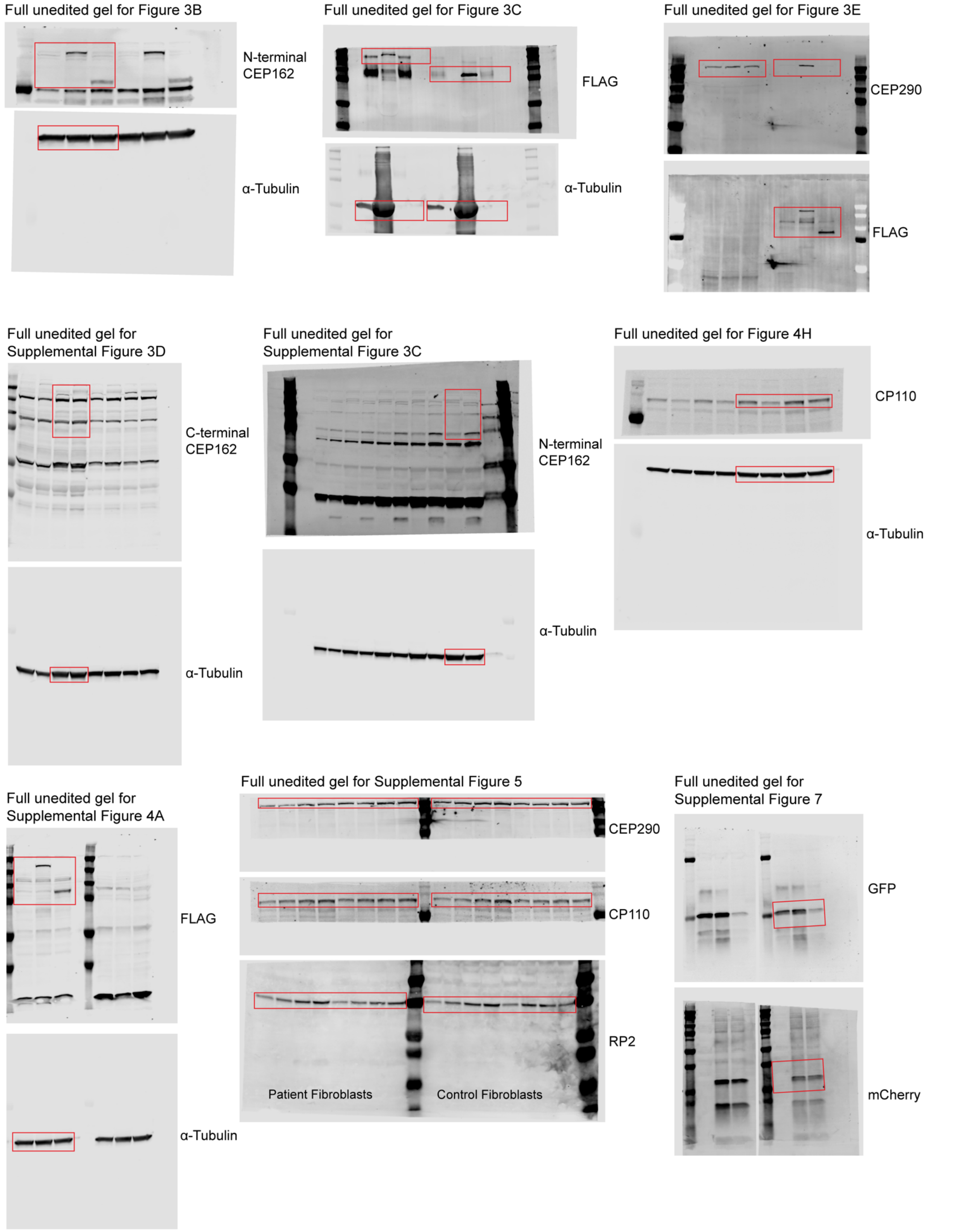

SUPPLEMENTAL FIGURE 9

Uncropped Western blot images. Red boxes delineate the cropped area of the blot shown in the indicated figures.

SUPPLEMENTARY METHODS

Assessment of nonsense-mediated decay (NMD)*.* To inhibit NMD, cultured fibroblasts (80-90% confluence) of the patient and a proband were incubated with anisomycin (1 µg/ml) for 6 h. RNA isolation and reverse transcription were performed as described previously (43). The expression change of CEP162 and an NMD control transcript after anisomycin treatment was analyzed by digital droplet PCR (ddPCR; QX200 Droplet Digital PCR System, Bio-Rad Laboratories; Hercules, CA) (for primers, see Supplemental Table 2).

Quantitative RT-PCR. Human control and patient cells were collected, RNA extracted using NucleoSpin RNA Kit (TaKaRa; Mountain View, CA) and adjusted to 50 ng/µl. cDNA was then synthesized from total RNA using iScript Advanced cDNA Synthesis Kit (Bio-Rad). Real time PCR was performed in a 10 µl reaction volume with 0.5 µl of cDNA using the iTaq Universal SYBR Green Supermix (Bio-Rad). Thermal cycling and SYBR detection were performed on a CFX96 Real Time System (Bio-Rad). A complete list of these primers can be found in Supplemental Table 2.

Immunoblotting. Cultured cells were grown to 80-85% confluency in a 15 cm dish, collected and gently homogenized with a pestle on ice in RIPA buffer (0.1% SDS, 0.5% sodium deoxycholate, 1% NP-40, 150 mM NaCl, 50 mM Tris-HCl, 5 mM EDTA, pH 8.0) with 1× cOmplete^{TM} protease inhibitor mixture (Millipore Sigma). Lysates were cleared at 10,000 ×g for 20 min at 4ºC and total protein concentration was measured using the RC DC Protein Assay kit (Bio-Rad). Lysates were subjected to SDS polyacrylamide gel electrophoresis using a AnykD Criterion TGX protein gel (Bio-Rad) and transferred to Immun-Blot LF PVDF (Bio-Rad). Western blotting was performed by blocking membranes in Intercept blocking buffer (LiCor Bioscience; Omaha, NE) for 1 hour at 22ºC, then incubating in the appropriate primary antibody diluted in 50% / 50% of Intercept / PBST overnight at 4ºC. Blots were then rinsed 3 times with PBST before incubating in the corresponding secondary donkey antibodies conjugated with Alexa Fluor 680 or 800 (LiCor Bioscience) in 50% / 50% / 0.02% of Intercept / PBST / SDS for 2 hours at 4ºC. Bands were visualized and quantified using the Odyssey CLx infrared imaging system (LiCor Bioscience). Images of the uncropped Western blots can be found in Supplemental Figure 9.

***CEP162* expression in human retinal single-cell transcriptional datasets**. Single-cell (sc) transcriptional datasets generated from adult human peripheral and foveal retina were mined for evaluating *CEP162* expression at the sc level (Supplemental Figure 8A). Expression matrices derived from pooling 3 donor retinas were retrieved and processed as described (2), resulting in 19,768 cells after quality control pre-processing. Following dimensionality reduction, embedding and clustering, *CEP162* expression was evaluated at sc-level using markers associated with major retina cell populations. All analyses were performed with SCANPY (v1.4.6) (3).

CEP162 immunofluorescence on human retina. Retinal samples of human donors were obtained from the Eye Bank of the Center of Ophthalmology, University of Cologne, Germany. The research followed the tenets of the Declaration of Helsinki. After dissection of the anterior segment, the remaining tissue included the posterior pole. Remaining vitreous humor was removed to obtain retinal tissue. For immunofluorescence analysis, horizontal retinal cryosections were fixed with 4% paraformaldehyde and rinsed with PBS. Sections were then rehydrated in PBS and preincubated with 1% dried milk in PBS and 0.01% Tween 20 to reduce nonspecific immunoreactivity. Overnight incubation with the primary anti-CEP162 antibody (HPA030172, Sigma-Aldrich, St. Louis, MO) was performed at 4 °C in PBS containing 2% BSA, 0.02% NaN3 and 0.1% Triton X-100. To estimate the specificity of the primary antibody, control stainings without primary antibody were performed in parallel. After washing in PBS, samples were labeled for 1 h at room temperature with the secondary anti-rabbit antibody conjugated to Alexa594 (red) (Dianova, Hamburg, Germany). Nuclei counter-staining was performed with 0.1 mg/ml DAPI (4′ ,6-diamidino-2-phenylindole) in PBS (Molecular Probes, Life Technologies, Frankfurt, Germany) for 10 min at room temperature. The cryosections were mounted with fluorescent mounting medium (Dako Cytomation, Hamburg, Germany) and viewed with a Zeiss Axio Imager. M2 fluorescence microscope equipped with ApoTome.2 (Carl

Zeiss, Jena, Germany). Microscopic pictures were analyzed with ZEN software (Carl Zeiss, Jena, Germany) (Supplemental Figure 8B-E).

CEP162 immunohistochemistry on human retina. Adult human retina was fixed in 10% neutral buffered formaldehyde and embedded in paraffin. Immunostaining of CEP162 was performed on 3-µm-thick sections using an automatic immunostainer (BenchMark Ultra, Ventana Medical Systems, Tucson, AZ). The rabbit polyclonal antibody anti-CEP162 (HPA030172, Sigma-Aldrich) was used, and visualization was achieved with the OptiView Amplification Kit (Ventana Medical Systems). Heat-induced epitope retrieval was performed using Cell Conditioning 2 (Ventana Medical Systems) (Supplemental Figure 8F).

References Supplements:

1 (previously 43):
2. Cowan CS, et al. Cell Types of the Human Retina and Its Organoids at Single-Cell Resolution. Cell. 2020 Sep 17;182(6):1623-1640.e34.
3. Wolf FA, et al. Large-scale single-cell gene expression data analysis. Genome Biol. 2018 Feb 6;19(1):15.
